# Splicing factor proline- and glutamine-rich (SFPQ) protein regulates platinum response in ovarian cancer-modulating SRSF2 activity

**DOI:** 10.1038/s41388-020-1292-6

**Published:** 2020-04-24

**Authors:** Ilenia Pellarin, Alessandra Dall’Acqua, Alice Gambelli, Ilenia Pellizzari, Sara D’Andrea, Maura Sonego, Ilaria Lorenzon, Monica Schiappacassi, Barbara Belletti, Gustavo Baldassarre

**Affiliations:** Division of Molecular Oncology, Centro di Riferimento Oncologico di Aviano (CRO) IRCCS, National Cancer Institute, 33081 Aviano, PN Italy

**Keywords:** Ovarian cancer, Apoptosis, RNA splicing

## Abstract

In epithelial ovarian cancer (EOC), response to platinum (PT)-based chemotherapy dictates subsequent treatments and predicts patients’ prognosis. Alternative splicing is often deregulated in human cancers and can be altered by chemotherapy. Whether and how changes in alternative splicing regulation could impact on the response of EOC to PT-based chemotherapy is still not clarified. We identified the splicing factor proline and glutamine rich (SFPQ) as a critical mediator of response to PT in an unbiased functional genomic screening in EOC cells and, using a large cohort of primary and recurrent EOC samples, we observed that it is frequently overexpressed in recurrent PT-treated samples and that its overexpression correlates with PT resistance. At mechanistic level, we show that, under PT treatment, SFPQ, in complex with p54^nrb^, binds and regulates the activity of the splicing factor SRSF2. SFPQ/p54^nrb^ complex decreases SRSF2 binding to *caspase-9* RNA, favoring the expression of its alternative spliced antiapoptotic form. As a consequence, SFPQ/p54^nrb^ protects cells from PT-induced death, eventually contributing to chemoresistance. Overall, our work unveils a previously unreported SFPQ/p54^nrb^/SRSF2 pathway that in EOC cells plays a central role in regulating alternative splicing and PT-induced apoptosis and that could result in the design of new possible ways of intervention to overcome PT resistance.

## Introduction

Epithelial ovarian cancer (EOC) is a relatively rare but highly lethal disease, representing the fifth leading cause of cancer death in women in the western world. The high death-to-incidence rate is mainly due to the late diagnosis, the peculiar way of metastatic dissemination and the appearance of chemoresistance [1, 2]. Gold standard therapy for EOC patients is cytoreductive surgery followed by platinum (PT) plus taxane-based chemotherapy. In particular, PT compounds (i.e., carboplatin and cisplatin) represent the backbone of first-line chemotherapy in EOC and also occupy an integral role in the management of relapsed ovarian cancer. The appearance of PT resistance dictates the choice of subsequent treatments and is predictive of poor prognosis [1, 2]. In EOC patients, de novo PT resistance is quite infrequent while acquired PT resistance is very common and has been linked to different mechanisms, including alteration in drug influx/efflux, increased DNA repair or tolerance to DNA lesions, defective activation, and/or execution of cell senescence/cell death [3, 4].

To identify new molecular alterations that could modify the sensitivity of EOC to PT, we performed a loss-of-function genomic approach for genes belonging to DNA repair, apoptosis, and TP53 pathways and identified SFPQ (splicing factor proline and glutamine rich) as a gene functionally involved in the response to PT.

SFPQ, also known as PSF (PTB-associated splicing factor), is a multifunctional nuclear protein first identified as a splicing factor, that participates to several cellular activities, including RNA transport, apoptosis, and DNA repair. Together with p54^nrb^ and PSPC1, it belongs to the well-conserved multifunctional DBHS (Drosophila behavior/human splicing) protein family that forms obligated homo- and heterodimers [5]. DBHS proteins are found in the nucleoplasm and, under various conditions, within sub-nuclear bodies termed paraspeckles, localized close to chromatin, or DNA damage foci [6]. SFPQ has both DNA- and RNA-binding domains and shares with its preferential binding partner p54^nrb^ most, but not all of its activities. Accordingly, SFPQ but not p54^nrb^ or PSPC1 seems to be essential for cell viability [7]. The precise mechanism by which SFPQ is required for cell growth and survival is not completely clarified, although some evidences indicate its involvement in the control of apoptosis [8–10]. A remarkable number of transcripts encoding for proteins involved in the apoptotic pathway are subjected to alternative splicing, eventually leading to the expression of proteins with opposite functions, either pro- or antiapoptotic [11, 12]. Changes in alternative splicing patterns were observed in association with acquired chemoresistance or under the selective pressure of PT treatment. For instance, it has been shown in breast cancer cells that cisplatin treatment induces the alternative splicing of more than 600 transcripts [13, 14]. Among those, several transcripts involved in apoptosis regulation, such as caspase-8, BCL2L1, Diablo, P73, Fas, were identified [14], suggesting that alternative transcripts could participate to the response of cancer cells to PT. In particular, it has been reported that basal and cisplatin-induced *caspase-8* and *-9* activation is regulated by the splicing factor SRSF2 (also known as SC35) [11, 15]. Accordingly, SRSF2 knock-down increased EOC cell survival under cisplatin exposure [16].

Here, we report that SFPQ is critically involved in EOC cell sensitivity to PT treatment and we identify a previously unknown activity of the SFPQ/p54^nrb^ complex that, via SRSF2 modulation, regulates caspase-9 alternative splicing and, eventually, leads to reduced PT-induced apoptosis.

## Results

### SFPQ modulates platinum (PT) response in epithelial ovarian cancer (EOC) cells

**Fig. 1:**
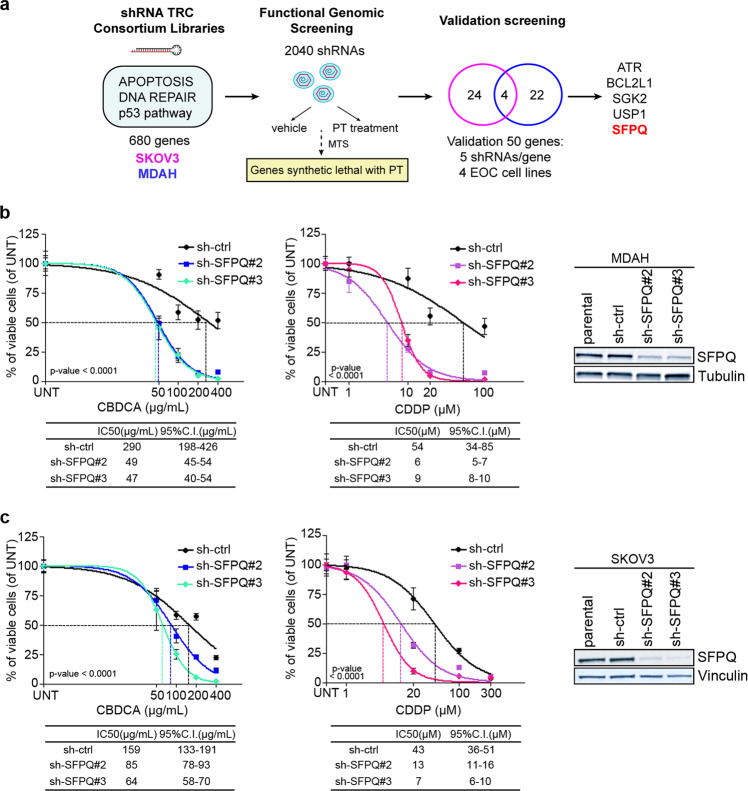
SFPQ modulates PT sensitivity in epithelial ovarian cancer cells. **a** Experimental design of the loss-of-function screening. Briefly MDAH and SKOV3 cells were transduced with 2040 shRNAs targeting 680 genes in 96-well plates in duplicate and treated or not with CBDCA for 16 h. Cell viability was evaluated by MTS assay, 24 h after the end of the treatment. Statistical analyses (*Z*-score and adjusted *p* value for multiple testing) identified 50 potential targets (22 in MDAH, 24 in SKOV3, and 4 in both cell lines). The 50 genes were then validated in a subsequent screening using five shRNAs/gene in four different cell lines. Validated genes were considered genes for which at least three shRNAs increased the sensitivity to CBDCA in at least three cell lines. Using these parameters we identified SFPQ (and other four genes). Nonlinear regression analyses of cell viability in MDAH (**b**) and SKOV3 cells (**c**). Cells were transduced with control shRNA (sh-ctrl) or two different SFPQ shRNAs (sh-SFPQ#2 and #3) and treated with increasing doses of CBDCA and CDDP for 16 h. The table shows the IC50 and the confidence interval (CI) of each condition. Data are expressed as percentage of viable cells compared with untreated cells and represent the mean (±SD) of three biological replicates. Fisher’s exact test was used to calculate the global *p* value reported in the graph. On the right western blot (WB) analysis reporting SFPQ expression in corresponding silenced cells. Tubulin and Vinculin were used as loading control.

To identify new putative mediators of PT response in EOC cells, we performed an shRNA-based loss-of-function screening to target 680 genes, belonging to apoptosis, TP53 and DNA repair pathways, which play key functions in EOC tumor progression and response to PT-based chemotherapy [17]. One TP53 mutated (MDAH-2774, hereafter MDAH) and one TP53-null (SKOV3) cell line were used, allowing us to identify genes involved in PT response both in the absence of p53 and in the presence of TP53 gain of function mutations, which represent the most common scenarios in EOC. Cells were transduced with the shRNA library and treated with sub-optimal doses of carboplatin (CBDCA) to obtain a ~20% reduction in cell viability (Fig. 1a). By this approach, we identified 50 candidate genes able to increase CBDCA activity when silenced, in at least one cell line. Validation screening by using five-specific shRNA/gene on four different EOC cell lines (i.e., MDAH, SKOV3, TOV112D, and OV-90), demonstrated that silencing of five genes, namely *ATR*, *BCL2L1* (also known as *Bcl-XL*), *SGK2*, *USP1*, and *SFPQ*, were able to significantly increase CBDCA response, in at least 3/4 tested cell lines. Since *ATR* and *Bcl-XL* involvement in PT response has been already well characterized, we decided to study the role of *USP1* [17], *SGK2* (manuscript in preparation) and *SFPQ*. Here, we report the data on the role of SFPQ in PT response. First, we validated the screening results using two-specific shRNAs against SFPQ. SFPQ knock-down significantly decreased the IC50, in MDAH and SKOV3 cells, to both CBDCA and Cisplatin (CDDP) (hereafter referred to as PT) (Fig. 1b, c). The similar results were obtained using two other independent EOC cell lines, OVCAR-3 and KURAMOCHI (Supplementary Fig. 1a, b), overall confirming that SFPQ played a role in the response to PT and that this role was independent from *BRCA1/2* status and the type of *TP53* alteration (Supplementary Fig. 1c).

### SFPQ expression increases after exposure to PT

**Fig. 2:**
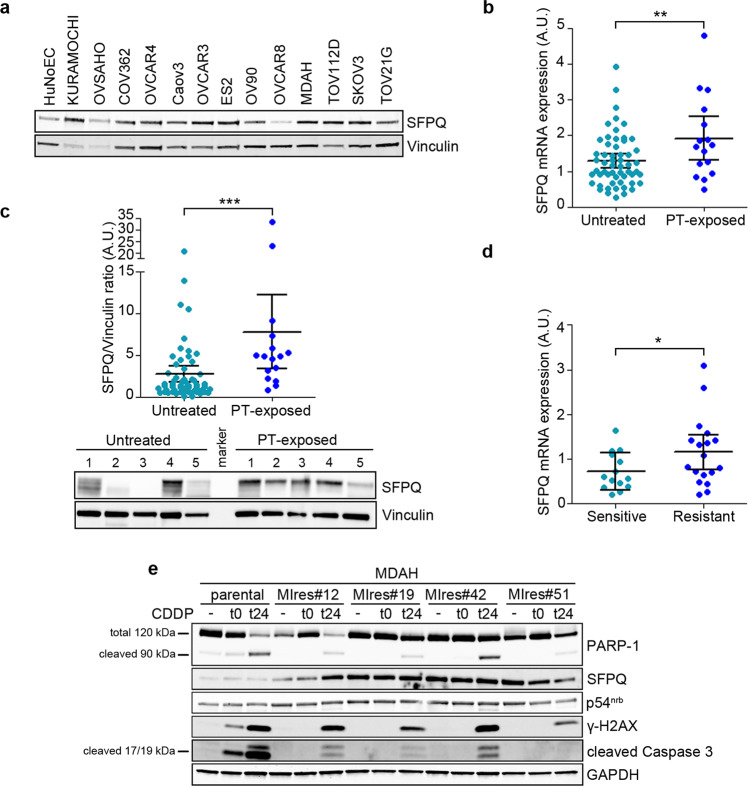
SFPQ is overexpressed in PT-resistant epithelial ovarian cancer. **a** WB analysis of SFPQ expression in normal human epithelial ovarian cells (HuNoEC) and in the indicated EOC cell lines. qRT-PCR (**b**) and WB analyses (**c**) reporting the normalized SFPQ expression in samples derived from untreated and PT-exposed (after neo-adjuvant chemotherapy or recurrent) EOC patients. A representative WB analysis of SFPQ expression is shown in (**c**, bottom panel). **d** qRT-PCR analysis reporting the normalized SFPQ mRNA expression in samples from PT-sensitive and PT-resistant EOC patients. **e** WB evaluating the expression of SFPQ and p54^nrb^ and of DNA damage (γ-H2AX) and apoptotic markers (PARP-1 and cleaved caspase-3) in parental and isogenic PT-resistant (MIres) MDAH cells treated or not with CDDP for 16 h (t0) and then released for 24 h (t24). In the figure statistical significance was determined by a two-tailed, unpaired Student’s *t* test. (*p < 0.05; **p < 0.01; ***p < 0.001). Vinculin or GAPDH expression was used as loading control.

SFPQ was generally more expressed in EOC cell lines compared with normal epithelial ovarian cells (i.e., HuNoEC). It was also widely expressed in a panel of primary EOC available in our Institute (Fig. 2a–c and Supplementary Table 1) [18, 19]. SFPQ expression (both at mRNA and protein levels) was significantly higher in EOC samples from patients that had previously received a PT treatment (samples collected after neo-adjuvant chemotherapy or recurrent EOC) (Fig. 2b, c) and in samples from PT-resistant (PT-Res) versus PT-sensitive EOC (Fig. 2d and Supplementary Table 2). Finally, the expression of SFPQ and of its preferential binding partner p54^nrb^ was higher in MDAH isogenic PT-Res cells, recently generated in our lab [20], compared with their parental counterpart (Fig. 2e). Next, using these cells in time course analysis of PT response [19–21], we evaluated the expression of SFPQ protein during the phases of PT-induced DNA damage and repair. The results showed that higher SFPQ expression in PT-Res cells correlated with decreased and delayed apoptosis and DNA damage response, as evidenced by PARP-1 and caspase-3 cleavage and γ-H2AX expression, respectively (Fig. 2e). Overall, these data suggest a possible role for SFPQ in the regulation of PT response.

### SFPQ knock-down increases basal and PT-induced apoptosis

**Fig. 3:**
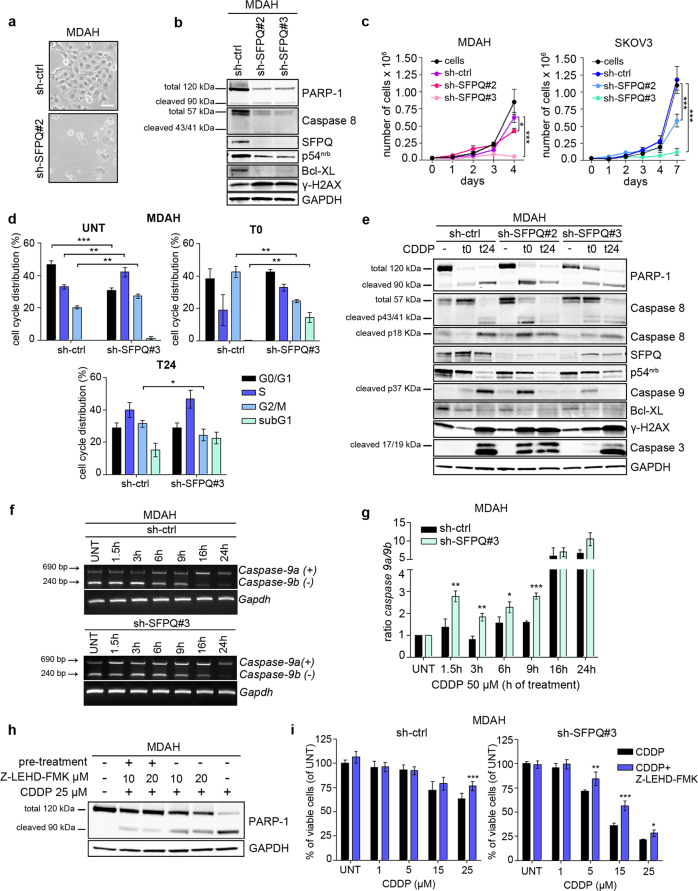
SFPQ knock-down increases basal and CDDP-induced cell death. **a** Typical phase contrast images (10× objective) of MDAH cells stable transduced with sh-ctrl or sh-SFPQ#2, after few days in culture. **b** WB analysis evaluating the expression of SFPQ and of DNA damage (γ-H2AX) and apoptotic markers (PARP-1, caspase-8, Bcl-XL) in cells silenced for SFPQ as in (**a**). **c** Growth curve of MDAH and SKOV3 cells transiently transduced with sh-ctrl and sh-SFPQ#2 and #3 as indicated. Cells growth was monitored by Trypan Blue Exclusion test. Data are the mean (±SD) of three biological replicates. **d** Cell cycle distribution of MDAH cells transiently transduced with the indicated shRNAs (sh-ctrl and sh-SFPQ#3) and analyzed by FACS 72 h after transduction in untreated and CDDP-treated conditions (CDDP 50 µM, T0 = 16 h treatment; T24 = 16 h treatment + 24 h release). The percentages of cells in each phase of the cell cycle are indicated. Data are the mean (±SD) of three biological replicates. **e** WB analysis evaluating the expression of PARP-1, caspase-8, -9, -3, γ-H2AX and Bcl-XL in MDAH cells transduced with sh-ctrl and sh-SFPQ#2 and #3 and treated as in (**d**). **f** Representative RT-PCR analyses of pro (+) and anti (−) apoptotic isoforms of *caspase-9* in MDAH cells transduced as indicated and treated with CDDP 50 µM for the indicated times. **g** Graphs reporting the quantification of the ratio between pro-/antiapoptotic isoforms of *caspase-9*, normalized on *Gapdh* expression and folded on untreated (UNT) condition. Data are the mean (±SD) of three independent experiments. **h** WB analysis evaluating PARP-1 cleavage in MDAH cells treated with CDDP 25 µM for 16 h, alone or in combination with Z-LEHD-FMK (caspase-9 inhibitor). **i** Dose-response curves evaluating cell viability of MDAH cells transiently transduced with sh-ctrl or SFPQ#3 shRNAs and treated with increasing doses of CDDP alone or in combination with 20 µM Z-LEHD-FMK. Data are expressed as percentage of viable cells compared with untreated cells and represent the mean (±SD) of three biological replicates. In the figure GAPDH was used as loading control and statistical significance was determined by a two-tailed, unpaired Student’s *t* test. (*p < 0.05; **p < 0.01; ***p < 0.001).

Long-term SFPQ knock-down (i.e., more than 1 week after antibiotic selection) induced massive cell death (Fig. 3a). As already reported by others [7], it was not feasible to generate stable SFPQ-silenced clones. This massive cell death was accompanied by increased ratio of cleaved/total PARP-1 and decreased expression of p54^nrb^, total caspase-8, and Bcl-XL (Fig. 3b), suggesting that the mechanism of apoptosis was involved. However, in transiently transduced MDAH and SKOV3 cells SFPQ knock-down decreased cell growth starting 3–4 days after transduction (Fig. 3c), leaving an opportunity for mechanistic investigation. Using this approach and FACS analysis we observed that SFPQ knock-down slightly but consistently increased the sub-G1 population both in basal condition and after PT treatment, accompanied by increased cell accumulation in S-phase, 24 h after PT removal (Fig. 3d and Supplementary Fig. 2a). At molecular level, the combination of SFPQ knock-down and PT treatment, in two different cell lines (i.e., MDAH and OVCAR-3), anticipated the activation of caspase-dependent cell death, compared with control cells. This was evidenced by the precocious expression of cleaved PARP-1, cleaved caspase-8, -9, and -3 (Fig. 3e and Supplementary Fig. 2b–d). Overall, these data supported the possibility that SFPQ directly participates to the regulation of basal and PT-induced apoptosis.

### SFPQ regulates the alternative splicing of *caspase-9* mRNA in PT-treated cells

To get more insights on the molecular pathway(s) that could drive apoptosis in SFPQ-silenced cells, we carried out a gene expression profiling (GEP) of MDAH and SKOV3 cells, silenced or not for SFPQ. Two KEGG-pathways were commonly enriched in both cell lines, namely the spliceosome and the ubiquitin-mediated proteolysis (Supplementary Fig. 3a, b and Supplementary Tables 3 and 4) and, given the involvement of SFPQ in splicing regulation, we decided to focus on the spliceosome. We observed that long-term SFPQ knock-down significantly increased the ratio of pro-/antiapoptotic forms of *caspase-9* RNAs, both in MDAH and SKOV3 cells (Supplementary Fig. 3c–e), supporting the possibility that SFPQ participates to the regulation of *caspase-9* alternative splicing, favoring the expression of antiapoptotic spliced forms.

We next verified if PT treatment induced changes in the splicing of apoptotic genes and how these changes preceded or accompanied cell death. In PT-treated MDAH cells, apoptosis was massively observed 24 h after PT removal, while it was minimal under PT treatment for up to 16 h (Fig. 3d, e and Supplementary Fig. 2a–d), as previously observed [18, 19]. We tested if PT treatment could alter the expression ratio of RNA of proapoptotic (marked with +) and antiapoptotic (marked with −) forms of caspases and BCL2L1, before the appearance of massive apoptosis. Time course analyses in MDAH cells showed that, starting from 16 h of PT treatment, a decrease of the antiapoptotic (−) forms of *caspase-8* and *-9* and a slight increase of the proapoptotic (+) ones, paralleled the induction of DNA damage (Supplementary Fig. 4a–c). In accord to its established role in mediating the intrinsic apoptotic pathway, these changes were particularly evident for *caspase-9* (Supplementary Fig. 4a, b). Consistent observations were made also for the expression of *BCL2L1*, with an increase of the *Bcl-X*_*S*_ (proapoptotic) and decrease of the *Bcl-X*_*L*_ (antiapoptotic) form (Supplementary Fig. 4a, b), demonstrating that PT treatment altered the alternative splicing of apoptotic genes in EOC cells.

More importantly, under PT treatment, knock-down of SFPQ in MDAH cells resulted in an increased expression of *caspase-9* (+) proapoptotic form, already visible after 1.5 h, while it had minor effects on *caspase-8* and *BCL2L1* splicing (Fig. 3f, g and Supplementary Fig. 5a–c). Accordingly, both the caspase-9-specific inhibitor Z-LEHD-FMK and the pan-caspase inhibitor Z-VAD-FMK, effectively prevented PARP-1 cleavage in PT-treated MDAH cells and increased cell survival of SFPQ-silenced MDAH cells (Fig. 3h, i and Supplementary Fig. 5d, e), confirming a central role for caspase-9 activation in PT-induced cell death when SFPQ was silenced.

### SFPQ regulates *caspase-9* splicing via SRSF2 regulation

Basal and PT-induced *caspase-8* and *-9* alternative splicing is mainly regulated by the splicing factor SRSF2 [11, 15], which has been reported to play an opposite effect compared with SFPQ in PT-treated EOC cells [16]. Accordingly, knock-down of SRSF2 in MDAH cells delayed the increase of the proapoptotic form of *caspase-9* (+) induced by PT, having minor effects on *BCL2L1* (Supplementary Fig. 6a, b). Based on these evidences, we better dissected the mechanism whereby SFPQ and SRSF2 controlled *caspase-9* splicing, in basal and PT-treated EOC cells.

**Fig. 4:**
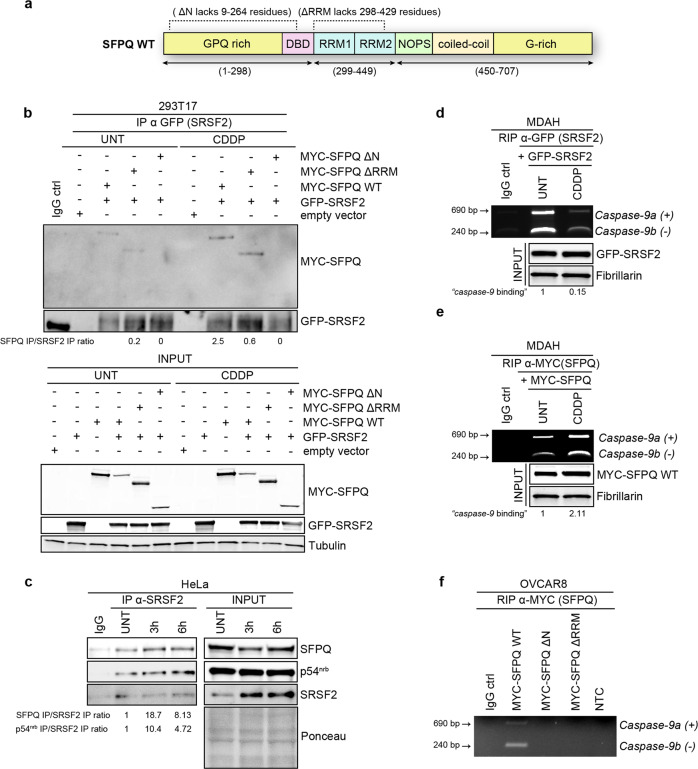
SFPQ binds *caspase-9* mRNA and interacts with SRSF2. **a** Schematic representation of SFPQ WT protein. RRM1 and RRM2, in light-blue indicate the RNA recognition motifs, NOPS (green) the NonA/paraspeckle domain and coiled coil (orange) the coiled coil domain. GPQ and G-rich (yellow) mark the low complexity regions and DBD (pink) the uncharacterized DNA-binding domain. SFPQ deletion mutants (ΔN and ΔRRM) used are indicated in the upper part of the scheme, reporting the correspondent amino-acidic deletion (SFPQ ΔN lacks 9–264 residues; SFPQ ΔRRM lacks 298–429 residues). **b** Co-IP analyses in 293T17 cells, transfected with the indicated vectors, treated or not with CDDP 50 µM for 3 h. Input reports the expression of the indicated protein in the lysates used for IPs experiments. Tubulin was used as loading control. Densitometric analysis of the IP ratio is reported under the blot. Anti-MYC Ab was used to reveal SFPQ expression. **c** Endogenous co-IP analyses of SFPQ, p54^nrb^, and SRSF2 in nuclear fraction of HeLa cells, in untreated (UNT) and CDDP-treated conditions (3 and 6 h of 50 µM CDDP). Lysates were IP with anti-SRSF2 antibody. Densitometric analysis of normalized SFPQ IP/SRSF2 IP and p54^nrb^ IP/SRSF2 IP expression is reported. RNA Immunoprecipitation analysis (RIP) in MDAH cells transfected with GFP-SRSF2 (**d**) or MYC-SFPQ WT (**e**) vectors and treated or not with CDDP 50 µM for 3 h. Samples were IP with the indicated antibodies. Densitometric analysis of normalized RIPs is reported under the blot. Overexpression of GFP-SRSF2 and MYC-SFPQ WT was confirmed by WB analysis. Fibrillarin was used as loading control. **f** RIP assay in OVCAR8 cells transfected with MYC-SFPQ WT and mutants constructs. Cells were treated with CDDP 50 µM for 3 h before harvesting. Samples were IP with anti-MYC antibody and amplified for *caspase-9* as described in (**e**).

First, we co-expressed SRSF2 and SFPQ, as full length or deleted isoforms (Fig. 4a) [22] in 293T17 cells then treated or not with PT. By co-immunoprecipitation assay we observed that SFPQ and SRSF2 co-precipitated under basal conditions and their binding was increased by PT treatment (Fig. 4b). The SFPQ-ΔN mutant lost the ability to bind SRSF2, indicating that this interaction requires the protein–protein interaction domain of SFPQ (Fig. 4b). SFPQ–SRSF2 interaction and its increase under PT treatment were confirmed using endogenous proteins from basal and PT-treated HeLa cells (Fig. 4c). Notably, also p54^nrb^, the preferential binding partner of SFPQ, co-precipitated with SRSF2 (Fig. 4c).

Using RNA immunoprecipitation (RIP) in MDAH cells treated or not with PT, we next demonstrated that both SRSF2 and SFPQ bound *caspase-9* RNA (Fig. 4d, e). Interestingly, PT treatment had opposite effects, on one side increasing SFPQ and the other decreasing SRSF2 binding to *caspase-9* RNA. (Fig. 4d, e). Using SFPQ-ΔN and -ΔRRM mutants or the SFPQ WT proteins we observed that SFPQ needs both domains to efficiently bind *caspase-9* RNA under PT treatment (Fig. 4f). These data support the possibility that this RNA-binding activity of SFPQ required the presence of other partners in the RNA/protein complex.

**Fig. 5:**
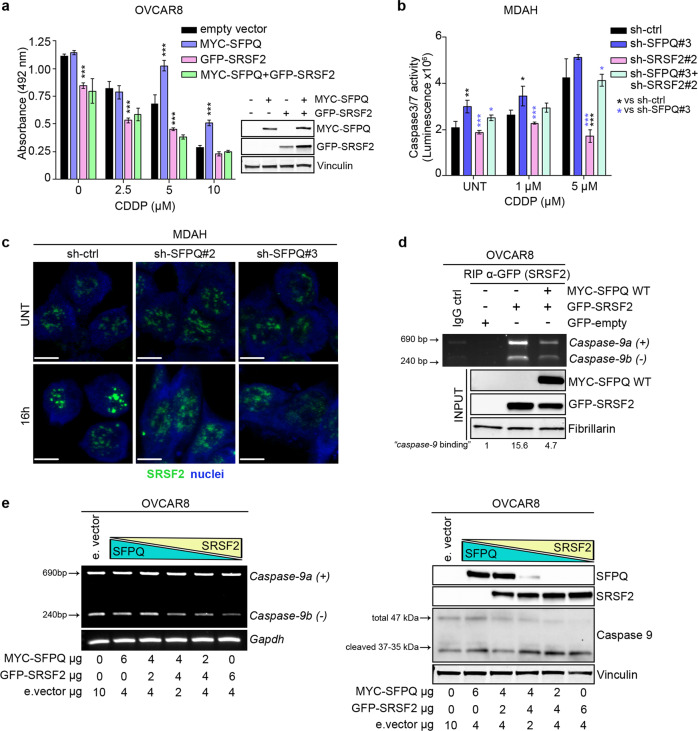
SFPQ–SRSF2 competitive binding influences caspase-9 RNA splicing regulation. **a** Dose-response curve of OVCAR8 cells overexpressing MYC-SFPQ WT and GFP-SRSF2 alone or in combination treated with increasing doses of CDDP. Data are expressed as absorbance of viable cells (*λ* = 492 nm) and represent the mean (±SD) of three biological replicates. On the right, the WB analysis reports MYC-SFPQ and GFP-SRSF2 expression in the used samples. **b** Dose-response analysis of MDAH cells transduced with the indicated shRNAs alone or in combination and then treated with increasing doses of CDDP. Caspase-3/7 activity was used as readout of apoptotic pathway activation. Data are expressed as luminescence (×10^6^) and represent the mean (±SD) of three biological replicates. **c** IF analysis evaluating SRSF2 (green) localization and expression in MDAH cells transduced with the indicated shRNAs left untreated or with CDDP 50 µM for 16 h. Nuclei were stained with propidium iodide (pseudocolored in blue); scale bar = 7 µm. **d** RIP analysis in OVCAR8 cells transfected with empty vector, GFP-SRSF2 and MYC-SFPQ WT alone or in combination and then treated with CDDP 50 µM for 3 h. Samples were IP with anti-GFP antibody, and the RNAs purified from the IP samples evaluated for *caspase-9* expression by RT-PCR. Overexpression of MYC-SFPQ WT and GFP-SRSF2 was confirmed by WB analysis. **e** Expression of *caspase-9* pro (+) and anti (−) apoptotic isoforms in OVCAR8 cells (left panel), transfected with decreasing (6–0 µg) and increasing (6–0 µg) doses of MYC-SFPQ WT and GFP-SRSF2, respectively. Cells were treated for 3 h with 50 µM CDDP. *Gapdh* was used as loading control. In the left panel WB confirming MYC-SFPQ and GFP-SRSF2 overexpression and caspase-9 expression and cleavage. In the figure Vinculin and Fibrillarin were used as loading control. Statistical significance was determined by a two-tailed, unpaired Student’s *t* test. (*p < 0.05; **p < 0.01; ***p < 0.001).

At biological level, we observed that SFPQ overexpression enhanced and SRSF2 overexpression reduced cell survival, in both basal and PT-treated OVCAR8 cells (Fig. 5a), used as a model of low SFPQ expression. When SFPQ and SRSF2 were co-transfected the effect of SRSF2 prevailed and cells were more prone to cell death (Fig. 5a). Accordingly, the increased PT-induced apoptosis observed in SFPQ-silenced cells was prevented by concomitant SRSF2 knock-down in MDAH cell (Fig. 5b and Supplementary Fig. 7a, b), indicating that SRSF2 was necessary in this process.

Using immunofluorescence analyses, we observed that SFPQ and SRSF2 co-localized in PT-treated MDAH cells in nuclear speckles (Supplementary Fig. 7c, d) and that SFPQ knock-down altered the PT-induced re-localization of SRSF2 (Fig. 5c). Moreover, RIP analyses in PT-treated OVCAR8 cells demonstrated that SFPQ reduced the ability of SRSF2 to bind *caspase-9* mRNA (Fig. 5d) and competition experiments, using increasing/decreasing concentrations of SFPQ and SRSF2 expressing vectors in OVCAR8 cells, showed that the antiapoptotic variant of *caspase-9* mRNA progressively decreased with the increased expression of SRSF2 and the reduced expression of SFPQ (Fig. 5e). Accordingly, the expression of *caspase-9* proapoptotic form paralleled the increased expression of SRSF2 protein (Fig. 5e).

### p54^nrb^ loss impairs SFPQ/SRSF2 interaction and influences *caspase-9* splicing

**Fig. 6:**
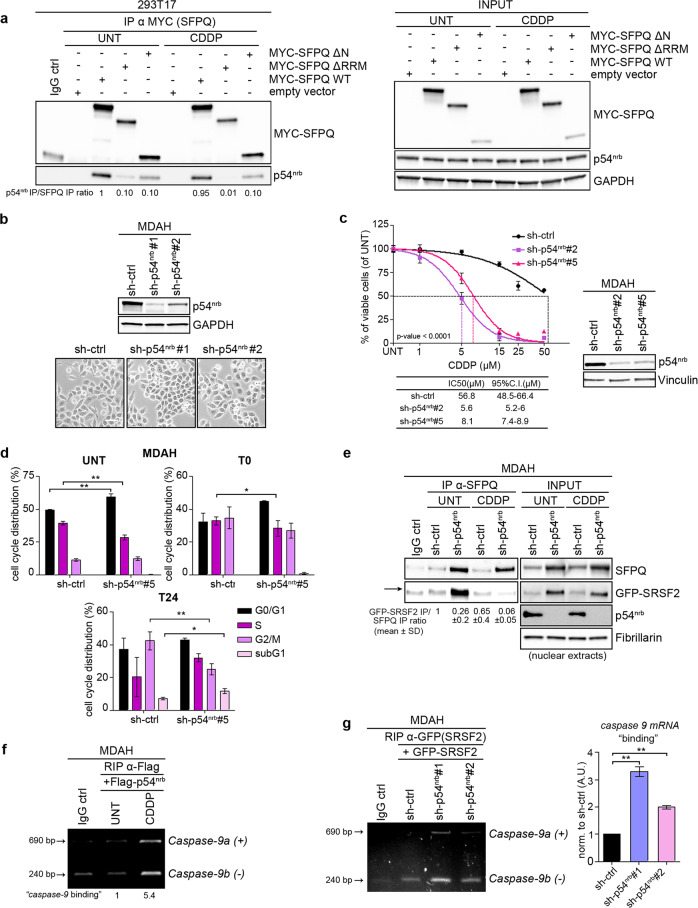
p54nrb knock-down influences SFPQ/SRSF2 interaction and caspase-9 mRNA splicing. **a** SFPQ-p54^nrb^ co-IP analyses in cells, transfected with the indicated vectors treated or not with CDDP 50 µM for 3 h. Input reports the expression of the indicated protein in the lysates used for IPs experiments. An anti-MYC Ab was used for the IP and to evaluate SFPQ expression. GAPDH was used as loading control. Densitometric analysis of normalized p54^nrb^/SFPQ interaction is reported under the blot. **b** Representative phase contrast images (10× objective) of MDAH cells stable transduced with sh-ctrl or sh-p54^nrb^ after puromycin selection. In the upper western blot the expression of p54^nrb^ in control and silenced pools is shown. **c** Nonlinear regression analyses evaluating cell viability of MDAH cells transiently transduced with sh-p54^nrb^ #2 and #5, as indicated, and treated with increasing doses of CDDP. The table reports the IC50 and the confidence interval (CI) of each condition. Data are expressed as percentage of viable cells compared with untreated cells and represent the mean (±SD) of three biological replicates. Fisher’s exact test was used to calculate the global *p* value reported in the graph. On the left, WB showing p54^nrb^ and levels in transduced cells. **d** Cell cycle distribution of MDAH cells transduced with sh-ctrl and sh-p54^nrb^#5, subjected to FACS analysis, 72 h after transduction in untreated and CDDP-treated conditions (CDDP 50 µM, t0 = 16 h treatment; t24 = 16 h treatment + 24 h release). The percentages of cells in each cycle phase are indicated. Data represent the mean (±SD) of three biological replicates. **e** Co-IP analysis of endogenous SFPQ and GFP-SRSF2 in MDAH cells described in (**b**) (pool sh-p54^nrb^#1), transiently transfected with GFP-SRSF2. Cells were treated with 50 µM CDDP for 3 h, and subjected to cytoplasmic/nuclear fractionation. IP was performed with anti-SFPQ antibody on nuclear extracts. Under the blot is reported the quantification of SFPQ–SRSF2 interaction in the different conditions that represents the mean (±SD) of three biological replicates. **f** RIP analysis in MDAH cells transfected with Flag-p54^nrb^ vector in untreated and treated conditions (CDDP 50 µM 3 h). Samples were IP with the indicated antibodies and the RNAs purified from the IP samples evaluated for *caspase-9* expression by RT-PCR. **g** RIP analysis in MDAH cells (described in (**b**)), transiently transfected with GFP-SRSF2 and treated with CDDP 50 µM for 3 h. Samples were IP with the indicated antibodies, and RNAs purified from the IP samples evaluated for *caspase-9* expression by RT-PCR (left panel). The right graph shows the densitometric analysis of the RIP and represents the expression of *caspase-9* mRNA normalized on sh-ctrl set at 1 as reference. Data represent the mean (±SD) of three biological replicates. In the figure, GAPDH, Vinculin or Fibrillarin were used as loading control. In (**d**) and (**g**) statistical significance was determined by a two-tailed, unpaired Student’s *t* test (*p < 0.05; **p < 0.01).

Our data indicated that SFPQ binding to *caspase-9* RNA could be mediated by another protein (Fig. 4f). The most common binding partner of SFPQ is p54^nrb^, which co-precipitated with both SFPQ and SRSF2, especially in PT-treated cells (Fig. 4c). Thus, we tested if p54^nrb^ also played a role in *caspase-9* mRNA splicing. First, we assessed that p54^nrb^ was generally well expressed in EOC cells and its expression slightly increased in PT-Res MDAH clones (Fig. 2e and Supplementary Fig. 8a). As expected, SFPQ WT and mutant proteins readily bound to endogenous p54^nrb^ and PT treatment did not modify the extent of their interaction in 293T17 cells (Fig. 6a). Differently to what observed in SFPQ-silenced cells (Fig. 3), MDAH cells stably silenced for p54^nrb^ displayed a growth rate that was only slightly reduced compared with their control counterpart (Fig. 6b). However, the downregulation of p54^nrb^ expression significantly increased PT-induced cell death (Fig. 6c). FACS analyses confirmed that in PT-treated MDAH cells, p54^nrb^ loss led to increased fraction of apoptotic cells (sub-G1 fraction) and reduced cell accumulation in G2/M-phase, particularly evident 24 h after PT removal, during the DNA repair phase (Fig. 6d and Supplementary Fig. 8b).

Interestingly, in p54^nrb^ silenced MDAH cells SFPQ binding to SRSF2 was reduced (Fig. 6e and Supplementary Fig. 8c, d), indicating that, in this context, p54^nrb^ regulated the reciprocal interaction between SFPQ and SRSF2. Further, the RIP assay demonstrated that p54^nrb^ bound the *caspase-9* RNA and that this binding was increased by PT treatment (Fig. 6f). Moreover, in MDAH cells stably silenced for p54^nrb^, SRSF2 bound with higher affinity *caspase-9* RNA (Fig. 6g), while its binding to *caspase-8* was unaltered compared with control cells (Supplementary Fig. 8e), again confirming that the expression of SFPQ/p54^nrb^ complex specifically regulated the splicing activity of SRSF2 on *caspase-9* RNA, in response to PT.

**Fig. 7:**
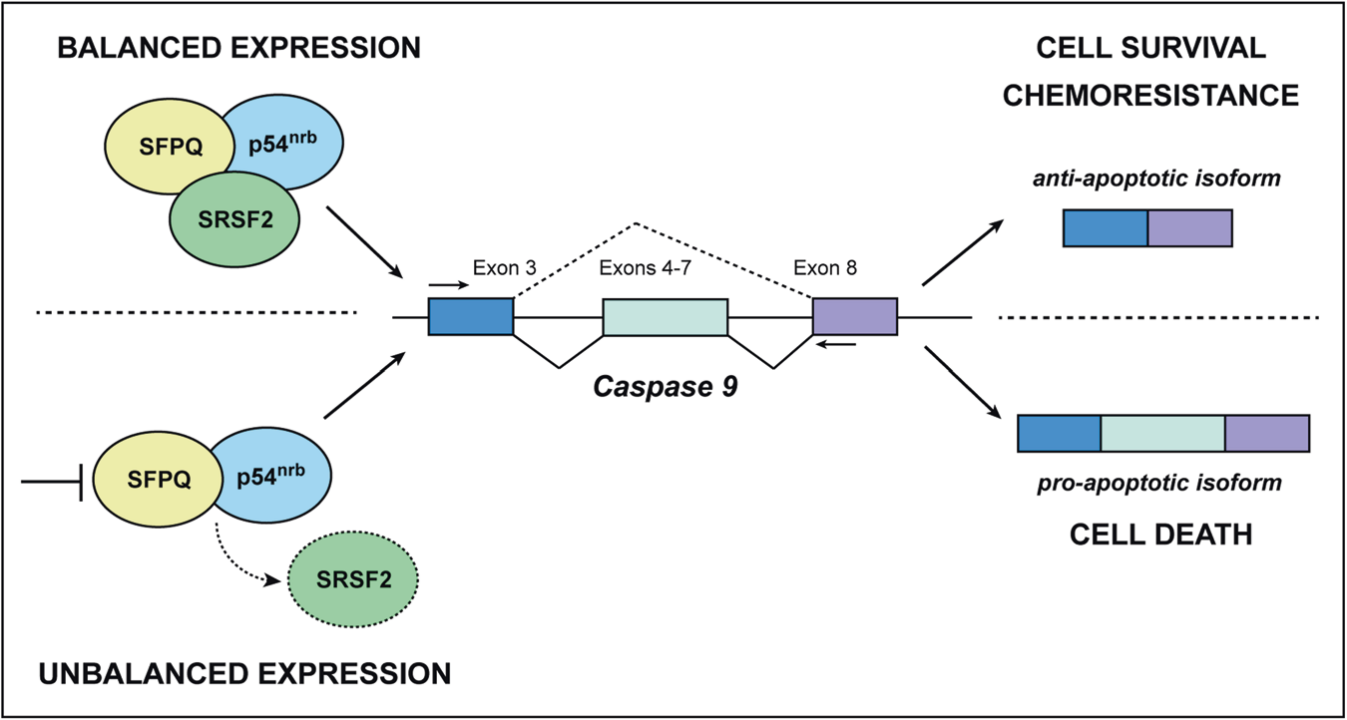
Proposed model for the role of SFPQ/p54nrb in PT response in EOC. SFPQ, in complex with p54^nrb^, binds and regulates the activity of the splicing factor SRSF2, already identified as a key factor in *caspase-9* alternative splicing regulation. In basal conditions, SFPQ/p54^nrb^ complex prevents/decreases SRSF2 binding to *caspase-9* RNA, favoring the expression of its antiapoptotic alternative spliced form and promoting cell survival. SFPQ/p54^nrb^ downregulation augment the binding of SRSF2 to caspase-9 RNA and leads to increased expression of *caspase-9* proapoptotic isoform, inducing cell death, in particular under platinum treatment. In this manner SFPQ/p54^nrb^ complex protect EOC cells from PT-induced death, eventually contributing to chemoresistance.

Overall, our work unveils a novel role for the SFPQ/p54^nrb^ complex in the regulation of *caspase-9* splicing. First, we observed that SFPQ/p54^nrb^ binds to SRSF2 and their relative expression levels are critical to determine whether exons 4–7 of *caspase-9* are retained or excluded from the mRNA, leading to different expression ratios of *caspase-9* pro- or antiapoptotic variants. The inhibition SFPQ/p54^nrb^ complex, combined with PT treatment, can increase the ability of SRSF2 to bind *caspase-9* RNA, thus enhancing its proapoptotic form and consequently cell death (Fig. 7).

## Discussion

Here, we describe a previously unknown pathway that links the SFPQ/p54^nrb^ complex to the regulation of PT-induced apoptosis, via the modulation of SRSF2 activity. Our data indicate that SFPQ binds to SRSF2 and counteracts its splicing activity on *caspase* RNAs, favoring the expression of antiapoptotic variants.

Several reports suggest that alternative splicing of specific genes, such as *BRCA1*, *BRCA2*, *BARD1*, or *ERCC1*, can impact on the response of cancer cells to PT and some studies also suggest that this correlates with PT resistance in human cancer [23–28]. Accordingly, it has been proposed that evaluation of alternative splicing of selected genes could serve as prognostic marker in ovarian cancer [29]. Our data demonstrate that the expression levels of SRSF2, SFPQ or p54^nrb^ impact on the sensitivity of ovarian cancer cells to PT-induced death and support the possibility that targeting alternative splicing, could also represent a promising therapeutic option to overcome PT resistance.

RNA sequencing and proteomic screenings have already demonstrated that treatment with PT impacts on the expression of genes involved in splicing regulation, suggesting again that this pathway represents an effective and dynamic way for cancer cells to respond to treatment-induced DNA damage [13, 14, 30, 31]. Indeed, our data suggest that PT treatment induced alternative splicing of apoptotic genes well before the appearance of cell death, supporting the possibility that it represents a rapid adaptive modification to survive.

The observations that SFPQ is highly expressed in primary EOC human samples and that its expression increased in samples from PT-exposed and PT-Res patients, support the possibility that SFPQ plays a role during cancer progression. Therefore, its evaluation as prognostic or predictive biomarker of PT response might merit further investigation in additional EOC cohorts. In line with our findings in EOC, very recent data have proposed a role for SFPQ in prostate cancer, where it promotes the expression of the androgen receptor variant 7, associated with castration resistance [32], supporting a role for SFPQ in promoting cancer-specific progression and resistance to therapy via the regulation of the alternative splicing.

We have previously reported a role for the splicing factor SRSF2 in the regulation of PT-induced alternative splicing in ovarian cancer and other groups also supported this possibility [15, 16]. Since SRSF2 is often overexpressed in ovarian cancer [33] and mutated in PT-treated ovarian cancer [16], we have focused on the interaction between SFPQ, p54^nrb^, and SRSF2. Our findings indicate that PT finely regulates the extent and the duration of this binding. Further studies will be necessary to precisely identify if and which PT-induced modification(s), either transcriptional or posttranscriptional, could alter this newly described interaction.

Overall, SFPQ plays pleiotropic roles in cells and acts differently when cells are under basal conditions or PT treated. Here, guided by the results of an unbiased GEP of SFPQ-silenced EOC cells (Supplementary Fig. 3), we focused on the role of SFPQ in the regulation of the spliceosome. We are aware that SFPQ could regulate other pathways like the ubiquitin-mediated proteolysis (enriched in two different EOC cell lines) that will need to be investigated further and this certainly represents a limitation of our study. Future studies will likely clarify if SFPQ exerts additional functions that also contribute to the survival of EOC, under basal and PT-treated conditions.

Indeed, several evidences suggest that SFPQ plays additional roles beyond the control of SRSF2-mediated regulation of *caspase-9* splicing. For instance, our data on the evaluation of PT-induced DNA damage (evaluated as γ-H2AX expression) suggested that SFPQ also participated to the control of DNA damage and repair. Further, we observed that caspase-9 inhibition reduced but did not completely reverted the PT-induced cell death observed in SFPQ-silenced cells (Fig. 3h, i), implying that other SFPQ-regulated mechanisms likely contributed to the response to PT in EOC cells. Accordingly, we observed an increased and anticipated expression of γ-H2AX in PT-treated cells that, conversely, was not changed in cells silenced for SRSF2 (Supplementary Fig. 9), suggesting that while SFPQ participated to the PT-induced DNA damage response, probably SRSF2 did not. This possibility is also supported by other data showing how the SFPQ/p54^nrb^ complex regulates the DNA damage response in vitro and in vivo in other models [7, 34, 35].

In our screening, we used shRNA libraries targeting genes involved in the apoptosis/DNA damage and *TP53* pathways and our experimental condition preferentially selected genes able to protect rather than sensitize to PT-induced death. It is therefore possible that we have missed the possibility to identify other targets or pathways that could be relevant for the regulation of PT response in EOC, or that some of the targets that have been identified could represent the consequence rather than the cause of EOC PT resistance. Also, it would be interesting to verify if the specific overexpression of caspase-9 antiapoptotic form could rescue the higher apoptosis induced by SFPQ/p54^nrb^ silencing. Despite these limitations, our work has unveiled a critical role for the SFPQ/p54^nrb^ complex in the response to PT in EOC cells. Our findings open the way for many new interesting investigations into the predictive and therapeutic implications of the alternative splicing in the processes of PT resistance and DNA damage response in ovarian cancer. Targeting the alternative splicing pathway is increasingly becoming a feasible opportunity to treat human diseases, including cancer [36–38]. Due to its crucial role in cell survival, the direct targeting of SFPQ seems not a pursuable strategy in patients. However, our data support the possibility that targeting p54^nrb^ could represent an alternative and more specific way to improve PT activity, especially in PT-Resistant EOC patients, for whom new therapeutic options are urgently needed.

## Materials and methods

### Primary EOC collection

Human EOC samples were collected by CRO institutional Biobank, immediately frozen and stored in liquid nitrogen until needed. Informed consent was obtained from all patients. The CRO Internal Review Board approved this study with the number CRO-IRB #05-2014.

### Loss-of-function screening

Loss-of-function screening was performed as described [17]. Briefly, the shRNAs library obtained by Sigma-Aldrich was used to perform the loss-of-function screening. On day one MDAH-2774 and SKOV3 cells were seeded in 96-well plates and the day after transduced with the specific shRNA or the control sh-ctrl. Seventy-two hours posttransduction cells were treated with CBDCA 140 µg/ml for 16 h. Cell viability was evaluated 24 h after the end of treatment using CellTiter 96 AQueous cell proliferation assay (MTS) kit (Promega).

### RNA immunoprecipitation (RIP)

EOC cells (~1 × 10^7^ for each condition), transfected with the indicated constructs, were lysed in complete RIP lysis buffer (nuclear isolation buffer, added with protease inhibitors cocktail (Roche) and RNAse (Promega)). After nuclear isolation and lysis of nuclear pellets, chromatin was sheared mechanically using sonication. The indicated (GFP/MYC/Flag or control IgG) antibodies were added to nuclear supernatant and incubated O/N at 4 °C. Protein A/G beads were added and incubated for 1 h 30 min at 4 °C. The immunoprecipitated samples were then centrifuged and washed with ice-cold RIP wash buffer, and the co-precipitated RNA was isolated by resuspending beads in TriZol (Invitrogen).

### Gene expression profiling

GEP were performed essentially as described [20, 39]. Briefly, 150 ng of total RNA were labeled with Cyanine (Cy)-3 dye andCy3-labeled RNA was hybridized using the Affymetrix Human Whole Genome (8 × 60 k) oligo-microarray platform (Agilent Technologies) and analyzed with an Agilent Microarray Scanner (Agilent Technologies) using the Agilent Feature Extraction Software 10.7.3 (Agilent Technologies). Microarray data have been deposited in NCBI Gene Expression Omnibus repository (#GSE131539) and could be accessed through the following link: https://www.ncbi.nlm.nih.gov/geo/query/acc.cgi?acc=GSE131539.

### Statistical analyses

All statistical analyses and graphs were performed using GraphPad PRISM software (version 6.0) using the most appropriate test, as specified in each figure. Independent groups are subjected to the comparison under the assumptions of normal distribution and equal variances. Data are presented as the mean ± SD of at least three biological replicates unless otherwise specified. Sample size for each experiment is indicated in the figure legend; no a priori criteria were established to predetermine sample size. Significant differences between means were determined with two-tailed Student’s *t* tests. For nonlinear regression analyses, Fisher’s exact test was used to calculate the global *p* value reported in the graph. SFPQ/SRSF2 co-localization (immunofluorescence analysis) was analyzed by Pearson correlation. The differences were defined as statistically significant (*p < 0.05), highly significant (**p < 0.01), and extremely significant (***p < 0.001). No samples were excluded from the analyses and no randomization or blinding was used.

“Materials and methods” are better described in Supporting Materials.

## Supplementary information

Supplemental Information
