# Supplementary material for: Splicing factor proline- and glutamine-rich (SFPQ) protein regulates platinum response in ovarian cancer-modulating SRSF2 activity
Source: Oncogene. 2020 Apr 24;39(22):4390–403. doi: 10.1038/s41388-020-1292-6 (PMC7253352; doi:10.1038/s41388-020-1292-6)
Supplement: Supplementary file 1 — Supplemental Information [file 41388_2020_1292_MOESM1_ESM.pdf]

Supplementary Information to Pellarin et al.

Splicing Factor Proline and Glutamine rich (SFPQ) protein regulates platinum response in ovarian cancer modulating SRSF2 activity

SUPPLEMENTARY FIGURES

a **b**

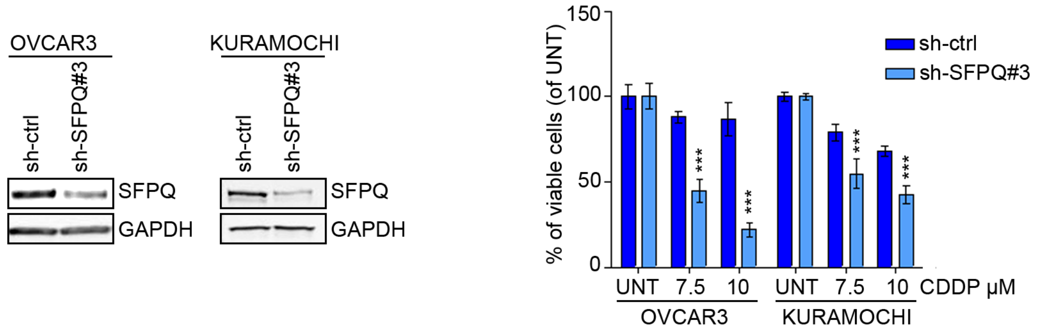

c

Cell line	p53	BRCA1	BRCA2	SFPQ expression	SFPQ silencing effect
MDAH	R273H	WT	1782fs	+++	✓
SKOV3	null	WT	WT	+++	✓
TOV112D	R175H	WT	WT	+++	✓
OV90	S215R	WT	WT	++	✓
OVCAR3	R248Q	WT	WT	+++	✓
KURAMOCHI	D281Y	WT	R2318*	+++	✓

Supplementary Fig. 1.SFPQ silencing influences response to cisplatin in EOC cells.

(**a**) Western blot analysis of SFPQ expression in OVCAR3 and KURAMOCHI cells, transduced with the indicated shRNAs. (**b**) Dose-response curve of the cells described in (**a**) and treated with increasing doses of CDDP. Data are expressed as percentage of viable cells respect to untreated cells and represent the mean (± SD) of 3 biological replicates. In the figure GAPDH was used as loading control. Statistical significance was determined by a two-tailed, unpaired Student's t-test. (*p<0.05; **p<0.01; ***p<0.001). (**c**) Table reporting p53, BRCA1/2 mutational status and SFPQ expression levels (based on WB analysis) of the indicated EOC cell lines. SFPQ silencing increase PT-induced cell death in all tested cell lines, (defined in table as SFPQ silencing effect).

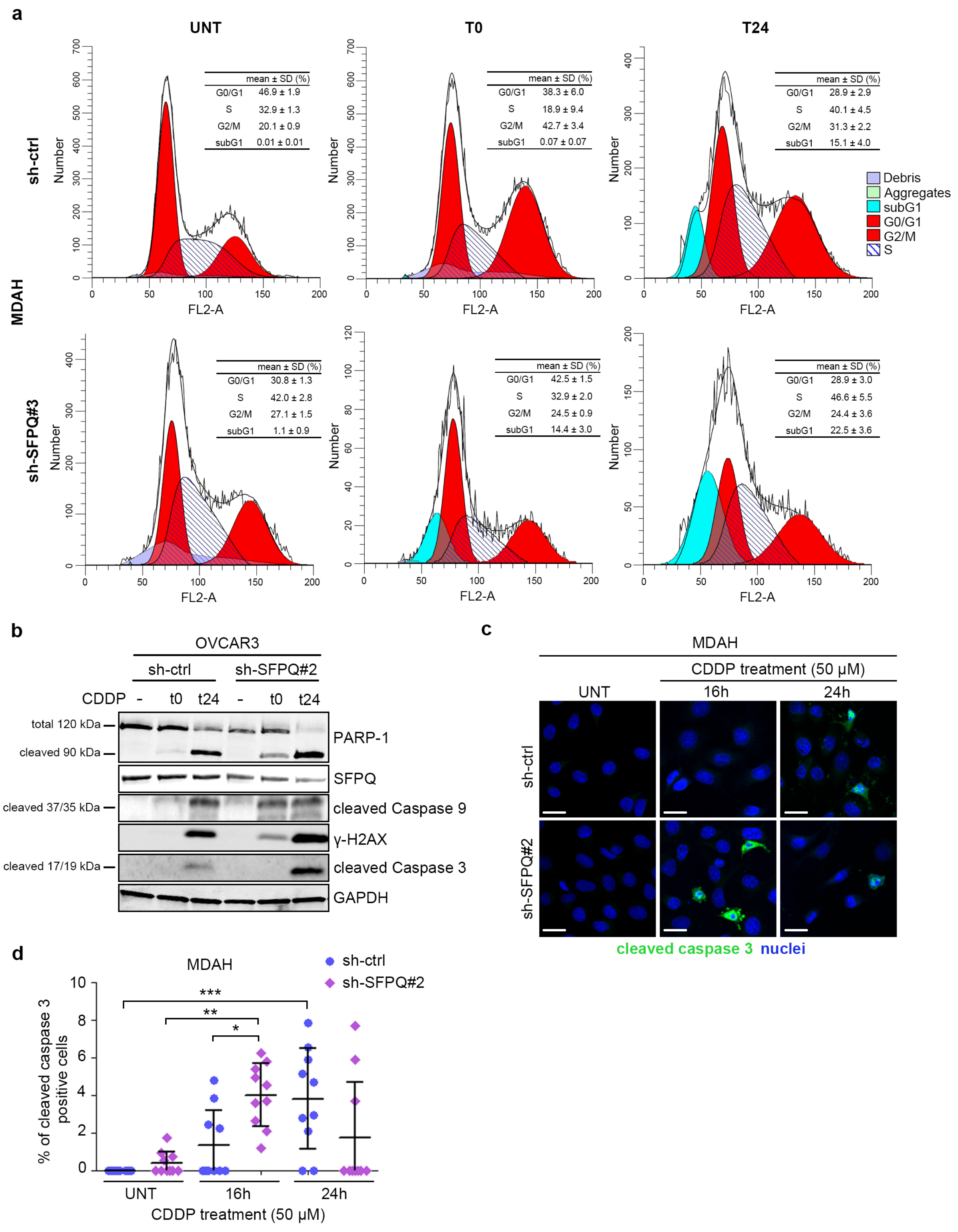

Supplementary Fig. 2.SFPQ silencing anticipates PT-induced apoptotic response.

(**a**) FACS analysis evaluating cell cycle distribution of untreated and CDDP-treated (50 μM) MDAH cells transduced with the indicated shRNAs (T0=16 hours treatment; T24= 16 hours treatment + 24 hours release). The propidium iodide (PI) stained cells were recorded by BD

FACScan™ flow cytometer and histograms were obtained with ModFit LT™ software. The inset table reports the cells percentage in G0/G1, S or G2/M phases, expressed as mean ± SD (n=3 biological replicates). (**b**) Western blot analysis showing the expression of SFPQ, PARP-1, cleaved caspase-9, -3 and γ-H2AX in OVCAR3 cells transduced with control (sh-ctrl) or SFPQ shRNA#2 and then treated with CDDP for 16 hours (t0) and released for 24 hours (t24). (**c**) Immunofluorescence (IF) analysis evaluating the expression of cleaved caspase-3 (green) in MDAH cells transiently silenced with sh-ctrl and sh-SFPQ#2 and treated as indicated; scale bar 20 μm. (**d**) Graph reports the percentage of cleaved caspase-3 positive cells/per field (± SD, n=10) in the cells described in (**c**).

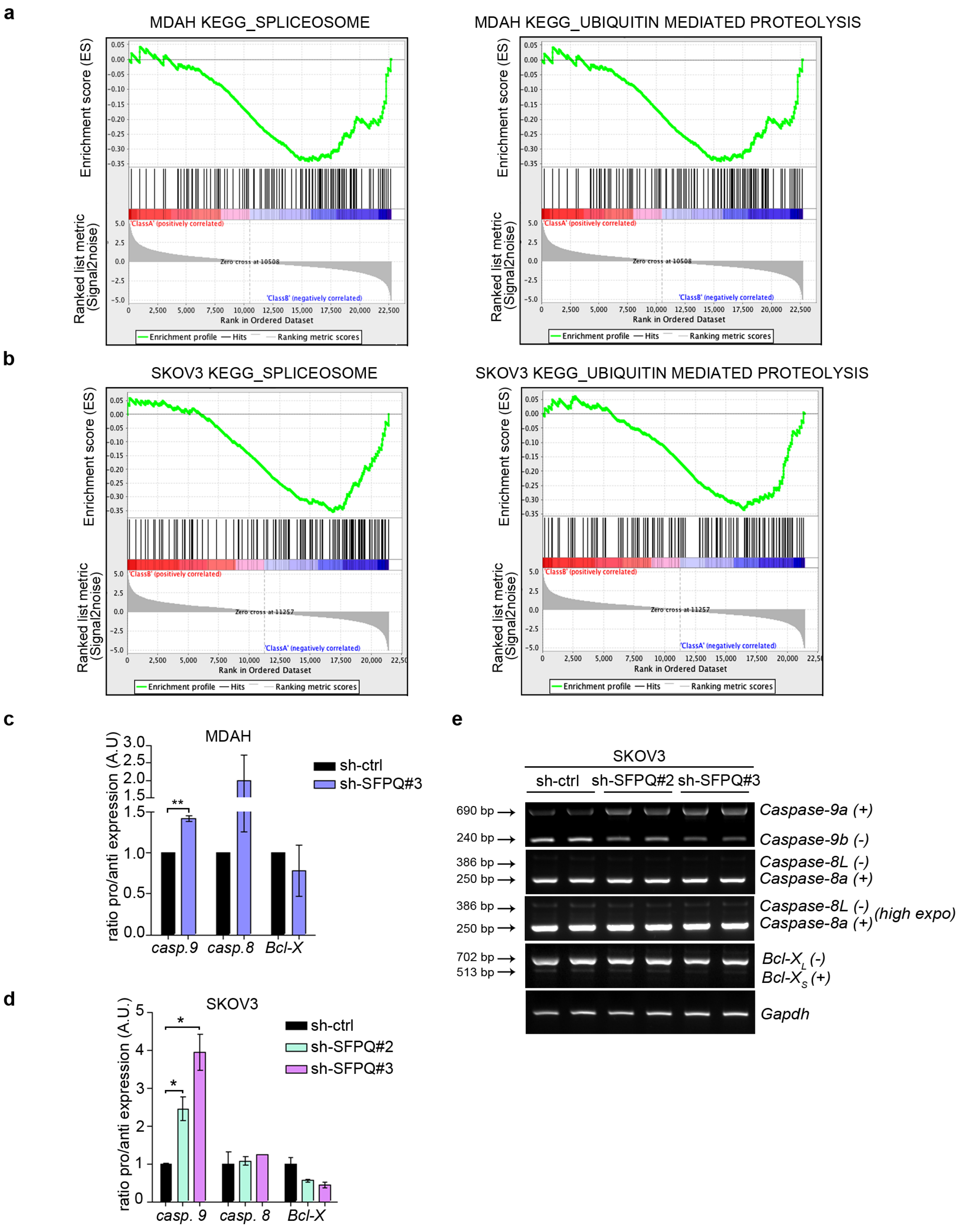

Supplementary Fig. 3.Spliceosome and Ubiquitin mediated proteolysis pathways are commonly modified by SFPQ silencing in EOC cells.

(**a**) and (**b**) Gene Set Enrichment Analysis (GSEA) of genes differentially expressed in EOC cells (MDAH and SKOV3) silenced for SFPQ respect to not silenced ones. A Signal2noise metric was used for ranking genes. Green curve denotes Enrichment Score. (**c**) and (**d**) Graph

reporting the quantification of the pro/anti isoforms ratio of *caspase-9*, *-8* and BCL2L1 genes in SFPQ silenced cells folded on sh-ctrl condition in MDAH (**c**) and SKOV3 (**d**) cells. Data are the mean ± SD of three independent experiments. Statistical significance was determined by a two-tailed, unpaired Student's t-test. *p<0.05 **p<0.01). (**e**) Representative RT-PCR analyses of pro (+) and anti (-) apoptotic isoforms of *caspase-9,-8* and BCL2L1 genes in SKOV3 cells transduced with the indicated shRNAs in duplicate.

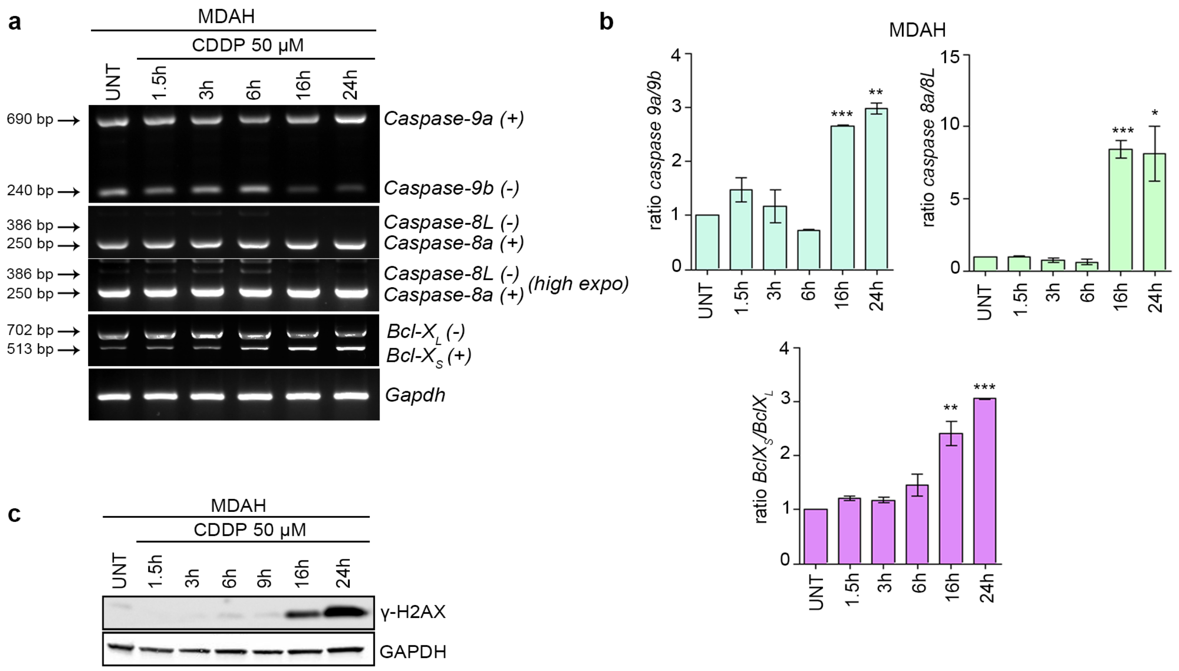

Supplementary Fig. 4. CDDP treatment induces the splicing of apoptotic genes in EOC cells.

(**a**) Representative RT-PCR analyses of pro (+) and anti (-) apoptotic isoforms of *caspase-9,-8* and BCL2L1 genes in MDAH cells treated with CDDP 50 µM at the indicated time points. *Gapdh* was used as loading control. (**b**) Graphs report the quantification of the ratio between pro/anti isoforms of *caspase-9*, *-8* and BCL2L1 genes, as described in (**a**), normalized on *Gapdh* expression and folded on untreated (UNT). Data are the mean (±SD) of three independent experiments. (**c**) WB showing γ-H2AX expression in time course experiment of MDAH cells treated with CDDP 50 µM for the indicated time points. GAPDH was used as loading control. Statistical significance was determined by a two-tailed, unpaired Student's t-test. (*p<0.05; **p<0.01; ***p<0.001).

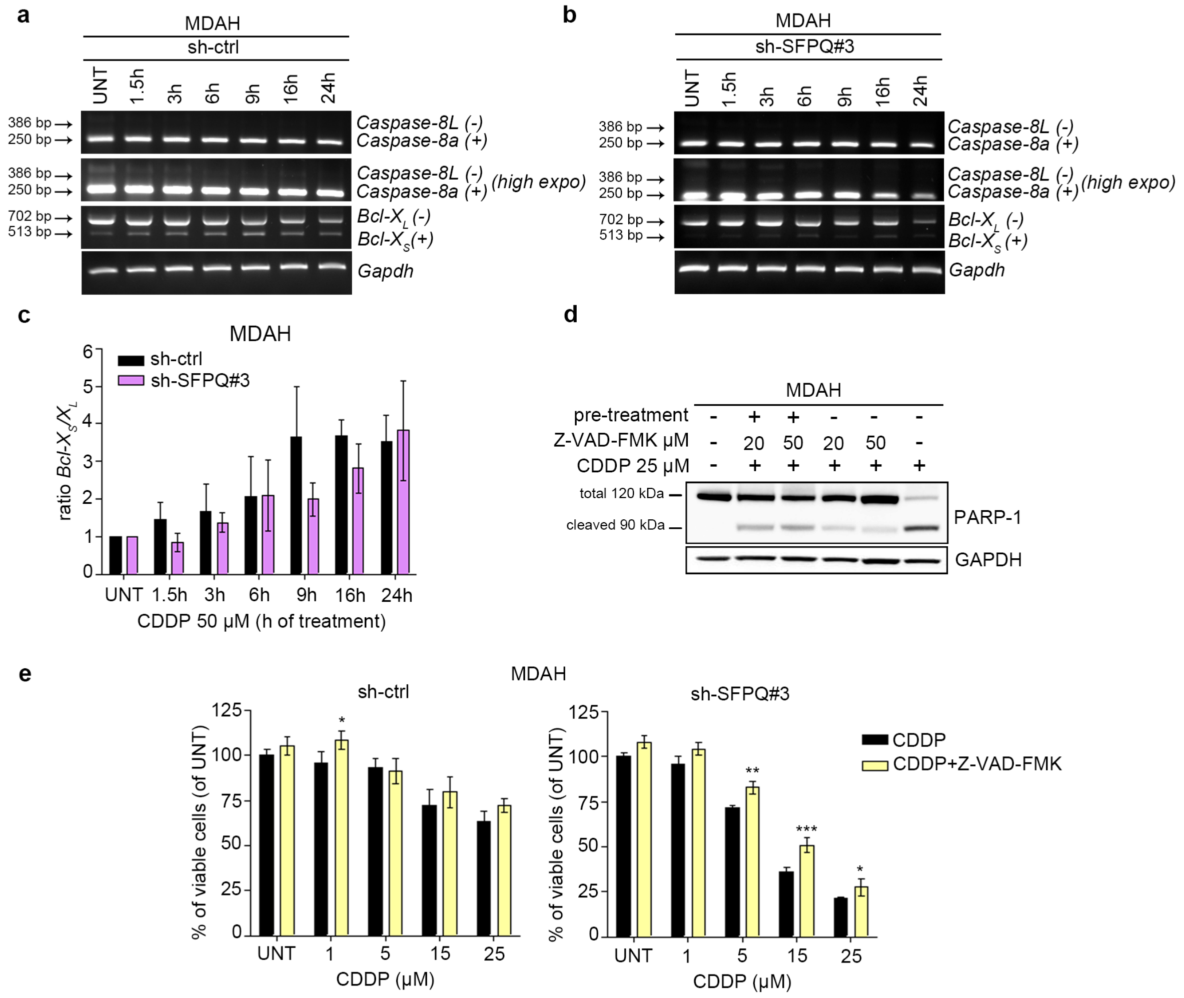

Supplementary Fig. 5. SFPQ regulates *caspase-9* alternative splicing in CDDP-treated cells.

(**a**) and (**b**) Representative RT-PCR analyses of pro (+) and anti (-) apoptotic isoforms of *caspase-8* and BCL2L1 genes in MDAH cells silenced for 72 hours with sh-ctrl (**a**) and sh-SFPQ#3 (**b**) and treated with CDDP 50 µM for the indicated times. (**c**) Graphs report the quantification of the ratio between pro/anti isoforms of BCL2L1 gene, normalized on *Gapdh* expression and folded on untreated (UNT) condition. (**d**) Western blot analysis evaluating PARP-1 cleavage in MDAH cells treated with CDDP 25 µM 16 hours, alone or in combination with Z-VAD-FMK (pan-caspase inhibitor). GAPDH was used as loading control. (**e**) Dose response curves evaluating cell viability of MDAH cells transiently transduced with sh-ctrl or SFPQ#3 shRNAs and treated with increasing doses of CDDP alone or with 20µM of Z-VAD-FMK. In (c) and (e) data represent the mean (± SD) of 3 biological replicates. Statistical significance was determined by a two-tailed, unpaired Student's t-test. (*p<0.05; **p<0.01; ***p<0.001).

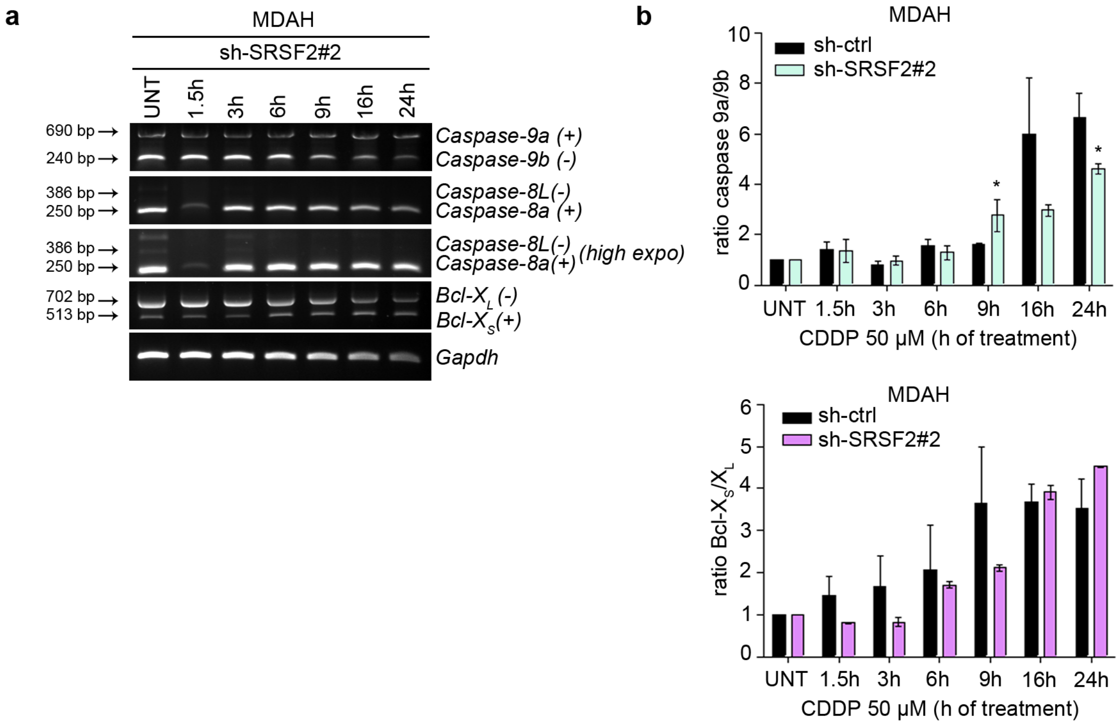

Supplementary Fig. 6.SRSF2 expression delayed the increase of the pro-apoptotic form of *caspase-9*, in CDDP-treated cells.

(**a**) Representative RT-PCR analyses of pro (+) and anti (-) apoptotic isoforms of *caspase-9*, -*8* and BCL2L1 genes in MDAH-2774 cells silenced for 72 hours with sh-SRSF2#2 and treated as reported in Supplementary Fig.5 a,b. (**b**) Graphs reporting the quantification of the ratio between pro/anti isoforms of *caspase-9* and BCL2L1 genes in SRSF2 silenced cells, normalized on *Gapdh* expression (loading control) and folded on untreated (UNT) condition. Data express the mean (±SD) of three independent experiments. Statistical significance was determined by a two-tailed, unpaired Student's t-test. (*p<0.05; **p<0.01; ***p<0.001).

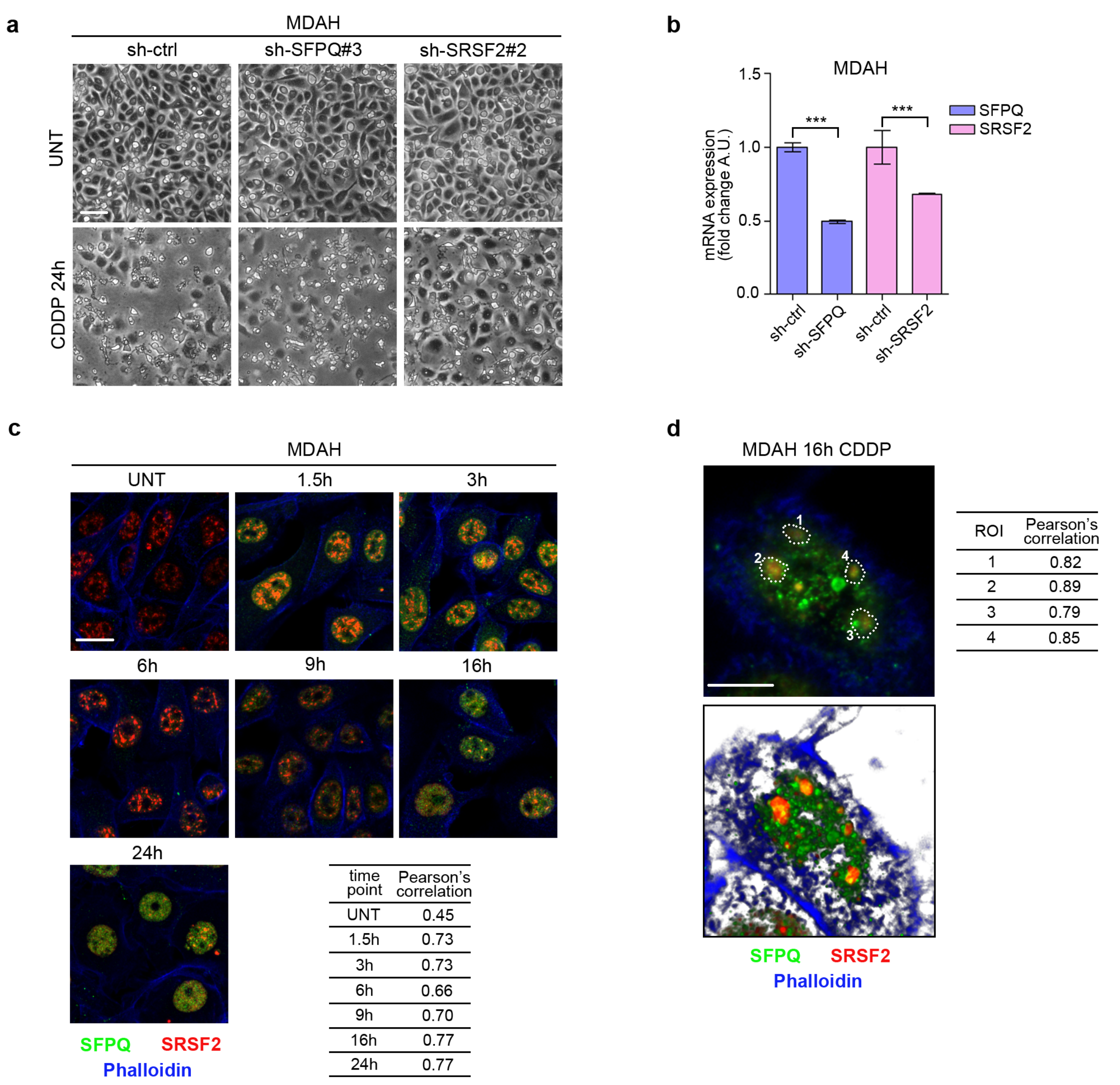

time point	Pearson's correlation
UNT	0.45
1.5h	0.73
3h	0.73
6h	0.66
9h	0.70
16h	0.77
24h	0.77

ROI	Pearson's correlation
1	0.82
2	0.89
3	0.79
4	0.85

Supplementary Fig. 7. SFPQ-SRSF2 binding and relative levels regulate CDDP-induced cell death.

(**a**) Typical phase contrast images (10X objective) of MDAH cells transiently transduced with sh-ctrl, sh-SFPQ#3 and sh-SRSF2#2 and then left untreated or treated with CDDP 50μM for 24 hours. (**b**) qRT-PCR analysis evaluating the expression of SFPQ and SRSF2 in MDAH cells silenced with the indicated shRNAs and described in (**a**). Data are expressed as fold change respect to sh-ctrl. Statistical significance was determined by a two-tailed, unpaired Student's t-test. (***$p<0.001$). (**c**) Immunofluorescence analysis evaluating SFPQ (green) and SRSF2 (red) expression and localization in MDAH cells, not treated (UNT) or treated with CDDP 50μM for the indicated time points. The inset table reports global Pearson's correlation value related to SFPQ/SRSF2 co-localization at each time point. Phalloidin

(pseudocolored in blue) was used to identify the cells in each field; scale bar= 20 µm. (**d**) Zoom of the immunofluorescence analysis described in (**c**) in which the nucleus of MDAH cells treated with CDDP 50 µM for 16 hours is magnified. In the upper panel: white dotted lined indicated the region of interest (ROI) chosen to more precisely evaluate SFPQ/SRSF2 co-localization. The corresponding value of Pearson's correlation is reported on the right table. In the lower panel the 3-D opacity projection image of the same area, obtained by Volocity software, is shown to better highlight co-localization (yellow spots). Scale bar= 8 µm.

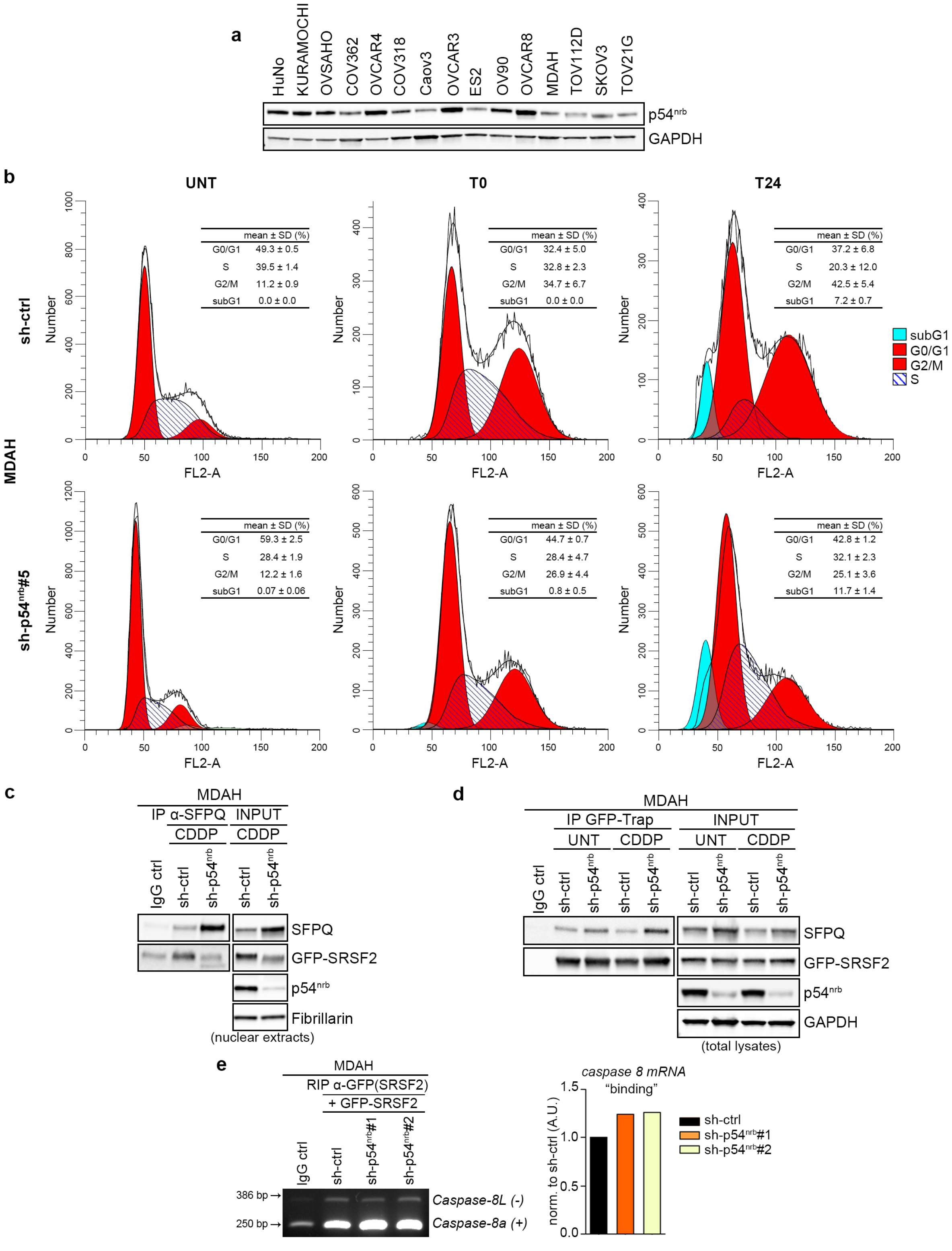

Supplementary Fig. 8. p54^{nrb} is expressed in EOC cells and contributes to the regulation of SFPQ/SRSF2 interaction.

(**a**) WB analysis of p54^{nrb} expression in normal Human Epithelial Ovarian cells (HuNoEC) and in the indicated Ovarian Cancer cell lines. GAPDH was used as loading control. (**b**)

FACS analysis evaluating cell cycle distribution of untreated and CDDP-treated (50 μM) MDAH cells transduced with the indicated shRNAs (T0=16 hours treatment; T24= 16 hours treatment + 24 hours release). The propidium iodide (PI) stained cells were recorded by BD FACScan™ flow cytometer and histograms were obtained with ModFit LT™ software. The inset table reports the cells percentage in G0/G1, S or G2/M phases, expressed as mean ± SD (n=3 biological replicates). (**c**) Co-IP analysis in MDAH stable pools (silenced with sh-ctrl and sh-p54^{nrb}), transiently transfected with GFP-SRFS2. IP was performed with anti-SFPQ antibody on nuclear extracts, from cells treated with CDDP 50 μM for 3 hours. (**d**) Co-IP analysis performed with GFP-trap system (SRSF2) antibody on total lysates, from MDAH cells described in (c) treated or not with CDDP (50μM for 3 hours) as indicated. (**e**) RIP analysis in p54^{nrb} stably silenced MDAH cells, transiently transfected with GFP-SRSF2 and treated with CDDP 50 μM for 3 hours. Samples were IP with anti-GFP (SRSF2) antibody, and RNAs purified from the IPs samples evaluated for *caspase-8* expression by RT-PCR (left panel). The right graph shows the densitometric analysis of the RIP and represents the expression of *caspase-8* mRNA normalized on sh-ctrl set at 1 as reference.

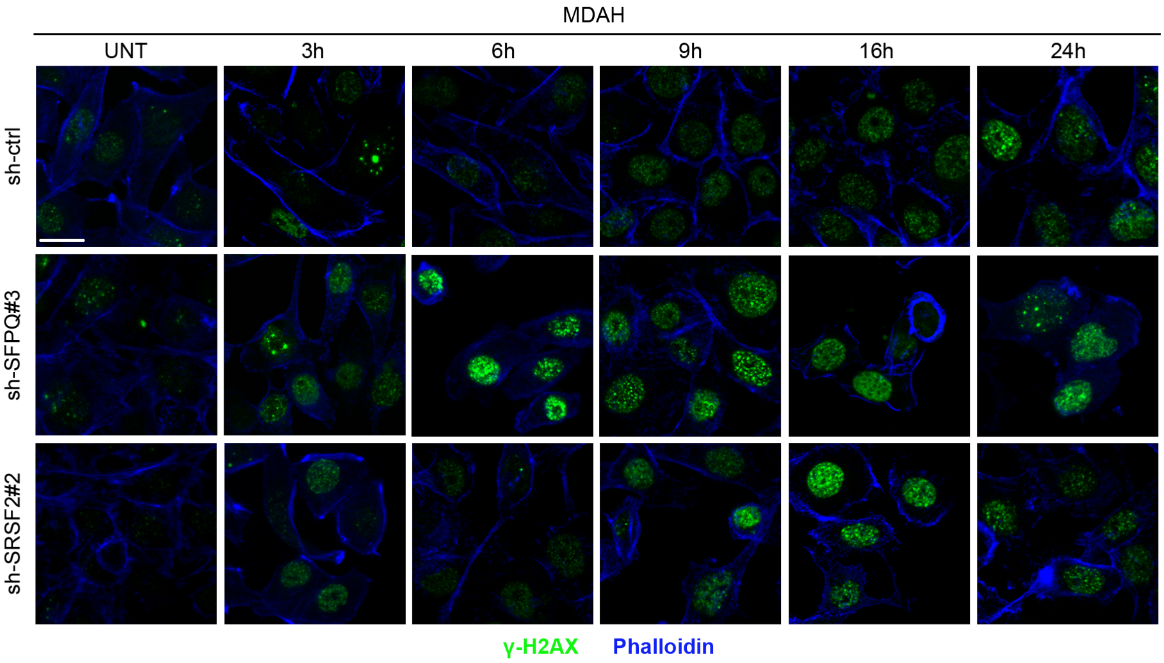

Supplementary Fig. 9. SFPQ but not SRSF2 silencing aanticipates the expression of the DNA damage marker γ -H2AX in CDDP-treated cells.

Immunofluorescence analysis evaluating γ-H2AX (green) expression in MDAH cells transiently transduced with sh-ctrl, sh-SFPQ#3 or sh-SRSF2#2 and then treated with CDDP 50μM for the indicated time points. In blue is shown the staining of F-Actin with Phalloidin; scale bar= 20 μm.

SUPPLEMENTARY TABLES

Supplementary Table S1

Characteristic of samples derived from ovarian cancer patients analyzed in Fig. 2b-c.

Serous Ovarian Cancer Samples	
**Characteristic**	**Value**
**Age-Year**	
Median	70
Range	48 -92
**Tumor Grade-no. (%)***	
Low (1-2)	3 (4.1)
High (3)	56 (77.7)
Not Available or Specified	13 (18.0)
**Tumor Stage-no. (%)****	
I or II	8 (11.1)
III or IV	56 (77.7)
Not Available or Specified	8 (11.1)
**Samples Type -no. (%)**	
Primary	56 (77.7)
Recurrence	16 (22.2)

* Include Borderline Ovarian Cancer (n=2) and unknown GX Ovarian cancer (n=6)

** According to FIGO

Supplementary Table S2

Characteristic of samples derived from PT-Sensitive and PT-Resistant ovarian cancer patients analyzed in Figure 2d.

Ovarian Cancer Samples		
**Characteristic**	**PT-Sensitive**	**PT-Resistant**[###]
**Age-Year**		
Median	67.5	71
Range	48-85	54-84
**Istotype-no. (%)**		
Serous	12 (85.7)*	14 (77.7)[#]
Endometrioid	1 (7.1)	0 (0)
Not Available or Specified	1 (7.1)	4 (22.2)[##]
**Tumor Grade-no. (%)**		
Low (1-2)	2 (14.2)	2 (11.1)
High (3)	5 (35.7)	12(66.6)
Not Available or Specified	7 (50)**	4 (22.2)
**Tumor Stage-no. (%)*****		
I or II	1 (7.1)	0 (0)
III or IV	8 (57.1)	14 (77.7)
Not Available or Specified	5 (35.7)	4 (22.2)
**Samples Type -no. (%)**		
Primary	12 (85.7)	15 (83.3)
Recurrence	2 (14.2)	3 (16.6)

EOC patients are defined PT-Resistant when experience disease relapse within 6 months from the end of the therapy and Refractory if they progress under the PT-treatment or within 3 months from the end of the therapy.

* Include Serous-Endometrioid Ovarian Cancer (n=1)
** Include Borderline Ovarian Cancer (n=1) and Unknown GX Ovarian cancer (n=2)
***According to FIGO
[#] Include Serous-Papillar Ovarian Cancer (n=2)
[##] Include Mucinous (n=2) and Undifferentiated (n=1) Ovarian Cancer
[###] Include Refractory Ovarian Cancer (n=4)

Supplementary Table S3

GSEA report for MDAH-2274 cells silenced or not for SFPQ.

Name of gene set	Genes in the gene set	Normalized enrichment score	Nominal p-value	FDR q-value
KEGG_AUTOIMMUNE THYROID DISEASE	25	-2,00	0,000	0,000
KEGG_NOTCH SIGNALING PATHWAY	43	-1,91	0,000	0,000
KEGG_MATURITY ONSET DIABETES OF THE YOUNG	20	-1,89	0,000	0,000
KEGG_RIBOSOME	84	-1,82	0,000	0,000
KEGG_GRAFT VERSUS HOST DISEASE	22	-1,82	0,000	0,000
KEGG_RNA POLYMERASE	28	-1,80	0,000	0,000
KEGG_BASAL TRANSCRIPTION FACTORS	31	-1,75	0,000	0,000
KEGG_CYTOSOLIC DNA SENSING PATHWAY	35	-1,73	0,000	0,000
KEGG_RNA DEGRADATION	56	-1,69	0,000	0,000
KEGG_ALLOGRAFT REJECTION	22	-1,66	0,000	0,010
KEGG_HEMATOPOIETC CELL LINEAGE	51	-1,63	0,000	0,019
KEGG_REGULATION OF AUTOPHAGY	22	-1,58	0,000	0,026
KEGG_COLORECTAL CANCER	59	-1,57	0,000	0,032
KEGG_CELL CYCLE	121	-1,57	0,000	0,030
KEGG_ACUTE MYELOID LEUKEMIA	55	-1,53	0,000	0,041
KEGG_CHRONIC MYELOID LEUKEMIA	71	1,52	0,000	0,058
**KEGG_UBIQUITIN MEDIATED PROTEOLYSIS**	130	-1,51	0,000	0,067
KEGG_ALDOSTERONE REGULATED SODIUM READSORPTION	32	-1,49	0,000	0,063
KEGG_NUCLEOTIDE EXCISION REPAIR	43	-1,47	0,000	0,071
KEGG_PIRYMIDINE METABOLISM	88	-1,43	0,000	0,099
KEGG_TYPE I DIABETES MELLITUS	26	-1,41	0,167	0,134
**KEGG_SPLICEOSOME**	124	-1,37	0,000	0,193
KEGG_MTOR SIGNALING PATHWAY	49	-1,36	0,25	0,208
KEGG_ENDOMETRIAL CANCER	48	-1,34	0,000	0,215
KEGG_VIRAL MYIOCARDITIS	51	-1,29	0,000	0,286

Gene sets modified in MDAH-2274 SFPQ silenced versus control-transduced cells according to Gene Set Enrichment Analysis (GSEA). FDR = False Discovery Rate.

Supplementary Table S4

GSEA report for SKOV3 cells silenced or not for SFPQ

Name of gene set	Genes in the gene set	Normalized enrichment score	Nominal p-value	FDR q-value
KEGG_OOCYTE MEIOSIS	98	1,82	0,000	0,000
KEGG_PENTOSE AND GLUCURONATE INTERCONVERS.	15	-1,76	0,000	0,061
KEGG_GAP JUNCTION	67	-1,72	0,000	0,0041
KEGG_VASCULAR SMOOTH MUSCLE CONTRACTION	78	-1,71	0,000	0,031
KEGG_PROTEASOME	41	-1,67	0,000	0,045
KEGG_MELANOMA	48	-1,66	0,000	0,032
KEGG_PROGESTERONE MEDIATED OOCYTE MATURATION	75	-1,63	0,000	0,032
KEGG_VALINE LEUCINE AND ISOLEUCINE DEGRADATION	40	-1,59	0,000	0,085
KEGG_CITRATE CYCLE TCA CYCLE	29	-1,58	0,000	0,087
**KEGG_SPLICEOSOME**	123	-1,57	0,000	0,090
**KEGG_UBIQUITIN MEDIATED PROTEOLYSIS**	129	-1,54	0,000	0,102
KEGG_LONG TERM DEPRESSION	46	-1,51	0,000	0,112
KEGG_WNT SIGNALING PATHWAY	119	-1,51	0,000	0,103
KEGG_DRUG METABOLISM OTHER ENZYMES	28	-1,50	0,000	0,129
KEGG_ADHERENS JUNCTION	69	-1,46	0,000	0,166
KEGG_TIGHT JUNCTION	100	-1,42	0,000	0,192
KEGG_REGULATION OF ACTIN CYTOSKELETON	161	-1,42	0,000	0,181
KEGG_CALCIUM SIGNALING PATHWAY	114	-1,38	0,000	0,246
KEGG_LONG TERM POTENTIATION	56	-1,34	0,000	0,268
KEGG_CELL CYCLE	117	-1,34	0,125	0,295
KEGG_VIBRIO CHOLERAE INFECTION	49	-1,34	0,000	0,281
KEGG_BASAL TRANSCRIPTION FACTORS	31	-1,33	0,250	0,294
KEGG_MELANOGENESIS	77	-1,31	0,000	0,307
KEGG_PORPHYRIN AND CHLOROPHYLL METABOLISM	28	-1,31	0,000	0,294
KEGG_STARCH AND SUCROSE METABOLISM	27	-1,30	0,333	0,282

Gene sets modified in SKOV3 SFPQ silenced versus control-transduced cells according to Gene Set Enrichment Analysis (GSEA). FDR = False Discovery Rate.

SUPPLEMENTARY MATERIALS AND METHODS

Cell lines

MDAH-2774 (CRL-10303), TOV-21G (CRL-11730), OV-90 (CRL-11732), SKOV-3 (HTB-77), NIH:OVCAR-3 (HTB-161) CAOV-3 (ATCC HTB-75) cells were obtained from American Type Culture Collection (ATCC); OVSAHO (JCRB1046) and KURAMOCHI (JCRB0098) cells were from JCRB Cell Bank; COV-362 [ECACC (European Collection of Authenticated Cell Cultures) 07071910] were from Sigma-Aldrich and OVCAR-8 and OVCAR-4 cells were from NCI Developmental Therapeutics Program Tumor Repository. All these cell lines were maintained in RPMI-1640 medium (Sigma-Aldrich). Human Epithelial Ovarian cells (HuNoEC, abm#T1074) were purchased from ABM (Applied Biological Materials) and cultured in Pri-grow I medium.Hela (ATCC, CCL-2), Human embryonic kidney (HEK) 293/T17 cells (ATCC, CRL-11268) used for overexpression approaches and 293FT cells (Invitrogen) used for lentivirus production, were grown in DMEM high-glucose (Sigma-Aldrich). All media were supplemented with 10% heat-inactivated fetal bovine serum (FBS) (Sigma-Aldrich) and 1% penicillin/streptomycin. Platinum-resistant cell lines were generated by treating MDAH cells for 2 h with a CDDP dose 10-fold higher than the IC50, followed by a recovery period. After 20 cycles of CDDP treatment, the resulting cell population was maintained in drug-free medium. All cell lines were grown in standard conditions at 37°C and 5% CO_2 and routinely authenticated in our lab using the Cell ID TM System (Promega) protocol and using Genemapper ID Ver 3.2.1 to identify DNA short tandem repeat profiles. Mycoplasma contamination was assessed every 15 days using the MycoAlert test (Lonza).

Primary EOC collection

Human EOC samples were collected by CRO institutional Biobank, immediately frozen and stored in liquid nitrogen until needed. Informed consent was obtained from all patients. The CRO Internal Review Board approved this study with the number CRO-IRB #05-2014.

Compounds and drug treatments

Carboplatin (CBDCA) and Cisplatin (CDDP) (TEVA Italia) were used in dose response-curves analyses, which are performed as described previously (1). Briefly EOC cells were seeded in 96-well culture plates and after 24 hours transduced with lentiviral shRNAs. 72 hours after transduction, plates were treated or not with CBDCA or CDDP for 16 hours at the indicated concentrations. Cell viability was determined 24 hours after treatment using the CellTiter 96 AQueous cell proliferation assay (MTS) kit (Promega, cat#G3582). For treatment with caspase-9 (Z-LEHD-FMK, cat#S7313) and pan-caspase (Z-VAD-FMK, cat#S7023) inhibitors (Selleckchem), cells were pre-treated for 2 hours with inhibitors at the indicated concentrations and then treated with CDDP increasing doses. Inhibitors were dissolved in dimethyl sulfoxide (DMSO) and stored at -20°C. Caspase activity was detected using the Caspase-Glo 3/7 assay system (Promega, cat#G8091).

Loss-of-function screening

Loss-of-function screening was performed as described [1]. Briefly, the shRNAs library obtained by Sigma-Aldrich, was used to perform the loss-of-function screening. On day one MDAH-2774 and SKOV3 cells were seeded in 96-well plates using a robotic liquid handling Hamilton’s MICROLAB STARlet. On day two cells were transduced with the specific sh-RNA or the control sh-ctrl. 72 hours post transduction cells were treated with CBDCA 140 µg/ml for 16 hours. Cell viability was evaluated 24 hours after the end of treatment using CellTiter 96 AQueous cell proliferation assay (MTS) kit (Promega, cat#G3582).

To identify shRNAs able to increase platinum sensitivity, log2 signal of the non-treated samples were compared to that of the treated to derive the log2 (non-treated/treated) ratios for each well in the twenty-two 96-well-plate among the two screening replicates. This ratio represents the changes in an shRNA's relative abundance between the non-treated and treated samples, with positive value indicating increased platinum sensitivity and negative value a decreased platinum sensitivity. We applied the Z-score normalization to account for plate-to-plate variation. Statistical analyses using a custom statistical package based on the Linear Models for Microarray data (Limma) method were used to correctly select the candidate shRNA. shRNAs that presented synthetic lethality or protective effect were ranked using a moderated t test statistic. To account for multiple testing problem Benjamini and Hochberg's method was applied to control the false discovery rate at a level of 5%. shRNAs that yielded synthetic lethality were filtered to identify genes that were targeted by multiple shRNAs.

This approach allowed selecting 50 genes potentially involved in the regulation of platinum sensitivity that were then validated in a second screening performed using five shRNAs for each gene in four different EOC cell lines, namely, MDAH, SKOV3, TOV112D and OV-90. Pre-specified conditions to validate a gene as able to affect PT-sensitivity were that at least 3/5 shRNAs displayed a significantly enhanced death after platinum treatment respect to controls in at least three different cell lines.

Vectors, transfections and recombinant viruses

pcDNA3 MYC-Tagged SFPQ WT, MYC-SFPQΔN, MYC-SFPQΔRRM and FLAG-Tagged $p54^{nrb}$ were obtained by Dr. Benjamin Blencowe through the Addgene Inc. consortium (Cambridge, Massachusetts) and described in [2]. The pEGFP-SRSF2 WT vector was kindly provided by Dr. Beatrice Eyminand and described in [3]. Plasmids were transfected using

FuGENE HD Transfection Reagent (Promega, cat#E2312) according to manufacturer's instructions.

For lentiviral particles production 293FT cells were co-trasfected, using standard calcium phosphate precipitation, with the lentiviral-based shRNA constructs (pLKO) and lentiviral system vectors pLP1, pLP2, and pVSV-G (Invitrogen), as described [4]. pLKO vectors encoding control and shRNAs for SFPQ, $p54^{nrb}$ and SRSF2 were purchased from Sigma Aldrich. Stable knock-down clones were selected by culturing cells in puromycin (Sigma Aldrich, cat# P9620, re-suspended in water) containing medium.

Growth curve and FACS analysis

MDAH and SKOV3 cells were plated in 6-well plates (50.000 cells/well) and transduced with sh-ctrl or sh-SFPQ#2 and #3. Viable cells were counted daily in triplicate, by trypan-blue dye exclusion method.

For FACS analysis, MDAH cells were plated in 100 mm dish and silenced with sh-ctrl, sh-SFPQ#3 or sh-$p54^{nrb}$#5. Cells were fixed in ice-cold 70% ethanol, washed twice in PBS1x and re-suspended in propidium iodide (50 µg /ml supplemented with 100 µg/ml RNase A in PBS1X). Stained cells were subjected to FACS analyses with FACScan instrument (BD Biosciences) and analyzed with ModFit LT™ software.

Preparation of cell lysates, Immunoblotting and Immunoprecipitation

Cell lysates were prepared using cold RIPA lysis buffer (150mM NaCl, 50mM Tris HCl [ph8], 1% Igepal, 0,5% NP40, 0,1% SDS) containing protease inhibitor cocktail (Roche), phosphatase inhibitors 1 mM Na_3VO_4, 10 mM NaF, and 1 mM DTT. Extraction of total proteins, Western Blot, and immunoprecipitations analyses were performed as described [1]. EGFP-SRSF2 protein transiently transfected in MDAH stable pools (silenced with sh-ctrl and sh-$p54^{nrb}$), was also IP using the GFP-Trap system (GFP-Trap®_A kit, Chromotek,#gtak-20),

a ready to use affinity resin for IP of GFP-fusion proteins, following manufacturer's instructions .

The following primary antibodies were used: Vinculin (1:1000, sc-73614 clone 7F9), MYC-tag (1:1000, sc-40 clone 9E10), Fibrillarin (1:1000, sc-25397 clone H-140), Bcl-XL (1:250, sc-56021 clone 7B2.5), PARP-1 (1:1000, sc-8007 clone F-2) were from Santa Cruz Biotechnology; Tubulin (1:5000, T9026 clone DM1A), SFPQ (rabbit 1:1000, AV40572 / mouse 1:1000 WH0006421M2), were from Sigma-Aldrich; caspase-8 (1:500, #9746 clone 1C12); caspase-9 (1:500, #9502), caspase-3 (1:500, #9661) were from Cell Signaling Technology; GAPDH (1:1000, CB1001 clone 6C5), γ-H2AX (1:1000, #05-636 clone JBW301) were from Calbiochem; SRSF2 (1:250 GTX82500) was from GeneTex; $p54^{nrb}$ (1:1000 #611279) was from BD-TL; GFP (1:500, #11814460001) was from Roche. Antibodies were visualized with appropriate horseradish peroxidase-conjugated secondary antibodies (GE Healthcare) for chemiluminescent detection (Clarity™ Western ECL Substrate cat#170-5061, Bio-Rad) or Alexa-conjugated secondary antibodies (Invitrogen, cat# A-21109 and A-32729) for Odyssey infrared detection (LI-COR Biosciences). Quantification of the immunoblots was done using the ImageLab software (Bio-Rad) or the Odyssey infrared imaging system (LI-COR Biosciences).

RNA extraction, RT-PCR and qRT-PCR

Total RNA was extracted using TriZol reagent (Invitrogen, cat#15596018) following manufacturer's instructions. Total RNA was quantified using the NanoDrop instrument (Thermo Fisher Scientific Inc., USA). RNA was retro-transcribed using random hexamers and the AMV Reverse Transcriptase (Promega, cat# M510F) or the Go-Script Reverse Trascription mix, Random primers (Promega, cat# A2801). cDNA were amplified for RT-PCR with GoTaq Green Master Mix protocol (Promega, cat# M712) and specific primers: *Caspase-8* (Forward, 5'-GGGATACTGTCTGATCATCAAC-3'; Reverse 5'-

GGAGAGGATACAGCAGATGAA-3') *Caspase-9* (Forward 5'-AGACCAGTGGACATTGGTTC-3'; Reverse 5'-GGTCCCTCCAGGAAACAAA-3') *Bcl-XL* (Forward 5'-ATGTCTCAGAGCAACCGGGA-3'; Reverse 5'-TCACTTCCGACTGAAGAGTG-3') *Gapdh* (Forward5'-GAAGGTGAAGGTCGGAGTC-3'; Reverse 5'- GAAGATGGTGATGGGATTTC-3'). Densitometric analyses of the PCR images were performed using ImageLab software (Bio-rad).

Absolute quantification was evaluated by qRT-PCR, using SYBR green dye-containing reaction buffer (Sso Fast Eva Green Supermix, cat# 172-5204, Biorad). Standard curves (10-fold dilution from 10^{-1} to 10^{-4} attomoles) were prepared and analyzed by qRT-PCR using the CFX-96 Real time System, C1000 Touch Thermal Cycler (Biorad). Primers used are: SFPQ (Forward 5'-AAGCACGCTGGTGCTAAGTA-3'; Reverse 5'-CAAACCCACTTTCACCCCCT-3') SDHA: (Forward 5'-AGAAGCCCTTTGAGGAGCA-3' Reverse 5'-CGATTACGGGTCTATATTC-3').

RNA Immuno Precipitation (RIP)

Hela, MDAH and OVCAR8 cells (~1x10^7 for each condition), transfected with the indicated constructs, were lysed on ice in complete RIP lysis buffer (Nuclear isolation buffer, added with protease inhibitors cocktail (Roche, cat# 11836145001) and RNAse (Promega, cat# N251A). After nuclear isolation and lysis of nuclear pellets, chromatin was sheared mechanically using sonication (6X pulses,10" each pulse 30% amplitude, Bandelin Sonoplus 3200). Indicated (GFP/MYC/Flag) or IgG control antibodies were added to nuclear supernatant (2 mg) and incubated O/N at 4°C with gentle rotation. Protein A/G beads (GE Healthcare, nProteinA Sepharose 4 Fast Flow, cat# 17-5280-01; Protein G Sepharose 4 Fast Flow, cat# 17-0618-01) were added and incubated for 1h 30 minutes at 4°C with gentle rotation. The immunoprecipitated samples were then centrifuged and washed 5 times with ice-cold RIP wash buffer. After the final wash, co-precipitated RNA was isolated by

resuspending beads in TriZol (Invitrogen, cat#15596018), followed by RNA extraction according to manufacturer's instructions. The cDNA was retro-transcribed from total RNA using the Go-Script transcriptase according to the manufacturers' instructions (Promega). PCR was then performed using specific primers. Densitometric analyses of the PCR images were performed using ImageLab software (Bio-rad). Nuclear isolation buffer: 1.28 M Sucrose, 40 mM Tris-HClph 7.5, 20 mM $MgCl_2$, 4% Triton X-100; RIP buffer: 150 mM KCl, 25 mM Tris-HClph 7.4, 5 mM EDTA, 0.5 mM DTT, 0.5% NP40, 100U/mL RNAse (Promega), protease inhibitors cocktail (Roche, cat# 11836145001).

Immunofluorescence

For immunofluorescence analyses cells plated on coverslips were fixed in PBS-4% paraformaldehyde (PFA) at room temperature (RT), blocked in PBS-1% bovine serum albumin (BSA) and stained as indicated with primary antibodies. Then samples were washed in PBS and incubated with secondary antibodies (1:200 dilution; Alexa-Fluor 488-, 633- or 546-conjugated anti-mouse or anti-rabbit antibodies;Invitrogen) for 1 hour at RT. Propidium Iodide (5 μg/ml + RNaseA) was used to visualize nuclei and Alexa-Fluor 647- or 546-Phalloidin (Invitrogen) for F-actin staining, 1:200 dilution. Coverslips were mounted with Mowiol/0.25% DABCO and analyzed using Leica Time Lapse AF6000LX workstation, the TCS-SP2 or the TCS-SP8 Confocal Systems (Leica Microsystems Heidelberg GmbH) interfaced with the Leica Confocal Software (LCS) or the Leica Application Suite (LAS) software. Collected images were analyzed using the Volocity (PerkinElmer) software. Primary antibodies used are: SFPQ (1:150, cat# AV40572 Sigma-Aldrich); SRSF2 (1:1000, cat# S4045 Sigma-Aldrich); γ-H2AX (1:200, #05-636, clone JBW301 Millipore); cleaved caspase-3 (1:300, #9661 Cell Signaling).

Gene Expression Profiling

Gene expression profiling (GEP) were performed essentially as described [5,6]. Briefly, total RNA samples derived from MDAH and SKOV3 cells (parental, sh-ctrl, sh-SFPQ#2, sh-SFPQ#3 samples for each cell line) was extracted using Trizol and purified in columns of RNEasy kit (Qiagen). GEP was performed with 150 ng total RNA labeled with Cyanine (Cy)-3 dye. Cy3-labeled RNA was hybridized using the Affymetrix Human Whole Genome (8x60k) oligo microarray platform (Agilent Technologies) and analyzed with an Agilent Microarray Scanner (Agilent Technologies) and with the Agilent Feature Extraction Software 10.7.3 (Agilent Technologies), as reported previously described [5,6]. After pre-processing and pre-filtering steps, the final dataset was subjected to supervised analyses using GeneSpring (Agilent Technologies). Gene expression profile results were visualized by hierarchical clustering applying Ward's method with Euclidean distance. Microarray data have been deposited in NCBI Gene Expression Omnibus (GEO) repository (#GSE131539).

Supplementary References

1 Sonego M, Pellarin I, Costa A, Rampioni G, Coan M, Kraut A *et al.* USP1 links platinum resistance to cancer cell dissemination by regulating Snail stability. Sci.Adv. 2019; **5**, eaav3235.

2 Rosonina E, Ip JYY, Calarco JA, Bakowski MA, Emili A, McCracken S *et al.* Role for PSF in mediating transcriptional activator-dependent stimulation of pre-mRNA processing in vivo. *Mol Cell Biol* 2005; **25**: 6734–6746.

3 Edmond V, Moysan E, Khochbin S, Matthias P, Brambilla C, Brambilla E *et al.* Acetylation and phosphorylation of SRSF2 control cell fate decision in response to cisplatin. *EMBO J* 2011; **30**: 510–523.

4 Dall'Acqua A, Sonego M, Pellizzari I, Pellarin I, Canzonieri V, D'Andrea S *et al.* CDK6 protects epithelial ovarian cancer from platinum-induced death via FOXO3 regulation. *EMBO Mol Med* 2017; **9**: 1415–1433.

5 Sonego M, Pellizzari I, Dall'Acqua A, Pivetta E, Lorenzon I, Benevol S *et al.* Common biological phenotypes characterize the acquisition of platinum-resistance in epithelial ovarian cancer cells. *Sci Rep* 2017; **7**: 7104.

6 Segatto I, Zompit MDM, Citron F, D'Andrea S, Vinciguerra GLR, Perin T *et al.* Stathmin Is Required for Normal Mouse Mammary Gland Development and Δ16HER2-Driven Tumorigenesis. *Cancer Res* 2019; **79**: 397–409.
